# The Multicopy Gene *Sly* Represses the Sex Chromosomes in the Male Mouse Germline after Meiosis

**DOI:** 10.1371/journal.pbio.1000244

**Published:** 2009-11-17

**Authors:** Julie Cocquet, Peter J. I. Ellis, Yasuhiro Yamauchi, Shantha K. Mahadevaiah, Nabeel A. Affara, Monika A. Ward, Paul S. Burgoyne

**Affiliations:** 1 Division of Stem Cell Biology and Developmental Genetics, Medical Research Council, National Institute for Medical Research, London, United Kingdom; 2 Department of Pathology, Mammalian Molecular Genetics Group, University of Cambridge, Cambridge, United Kingdom; 3 Institute for Biogenesis Research, University of Hawaii Medical School, Honolulu, Hawaii, United States of America; MRC Human Genetics Unit, United Kingdom

## Abstract

Studies of mice with Y chromosome long arm deficiencies suggest that the male-specific region (MSYq) encodes information required for sperm differentiation and postmeiotic sex chromatin repression (PSCR). Several genes have been identified on MSYq, but because they are present in more than 40 copies each, their functions cannot be investigated using traditional gene targeting. Here, we generate transgenic mice producing small interfering RNAs that specifically target the transcripts of the MSYq-encoded multicopy gene *Sly* (*Sycp3*-*like Y-linked*). Microarray analyses performed on these *Sly*-deficient males and on MSYq-deficient males show a remarkable up-regulation of sex chromosome genes in spermatids. SLY protein colocalizes with the X and Y chromatin in spermatids of normal males, and *Sly* deficiency leads to defective repressive marks on the sex chromatin, such as reduced levels of the heterochromatin protein CBX1 and of histone H3 methylated at lysine 9. *Sly*-deficient mice, just like MSYq-deficient mice, have severe impairment of sperm differentiation and are near sterile. We propose that their spermiogenesis phenotype is a consequence of the change in spermatid gene expression following *Sly* deficiency. To our knowledge, this is the first successful targeted disruption of the function of a multicopy gene (or of any Y gene). It shows that SLY has a predominant role in PSCR, either via direct interaction with the spermatid sex chromatin or via interaction with sex chromatin protein partners. *Sly* deficiency is the major underlying cause of the spectrum of anomalies identified 17 y ago in MSYq-deficient males. Our results also suggest that the expansion of sex-linked spermatid-expressed genes in mouse is a consequence of the enhancement of PSCR that accompanies *Sly* amplification.

## Abstract 2

Small-interfering RNAs have been used to disrupt the function of the more than 100 copies of the *Sly* gene on the mouse Y chromosome, leading to defective sex chromosome repression during spermatid differentiation and, as a consequence, sperm malformations and near-sterility.

## Author Summary

During meiosis in the male mouse, the X and Y chromosomes are transcriptionally silenced, and retain a significant degree of repression after meiosis. Postmeiotically, X and Y chromosome–encoded genes are consequently expressed at a low level, with the exception of genes present in many copies, which can achieve a higher level of expression. Gene amplification is a notable feature of the X and Y chromosomes, and it has been proposed that this serves to compensate for the postmeiotic repression. The long arm of the mouse Y chromosome (MSYq) has multicopy genes organized in clusters over several megabases. On the basis of analysis of mice carrying MSYq deletions, we proposed that MSYq encodes genetic information that is crucial for postmeiotic repression of the sex chromosomes and for sperm differentiation. The gene(s) responsible for these functions were, however, unknown. In this study, using transgenically delivered small interfering RNA, we disrupted the function of *Sly*, a gene that is present in more than 100 copies on MSYq. *Sly*-deficient males have major sperm differentiation problems together with a remarkable postmeiotic derepression of genes encoded on the X and Y chromosomes. Furthermore, the epigenetic modifications normally associated with sex chromosome repression are altered. Our data thus show that the SLY protein is required to mediate postmeiotic repression of the X and Y chromosomes. It is likely that the sperm differentiation problems in *Sly*-deficient males are largely a consequence of the derepression of the sex chromosomes in spermatids. We propose that the postmeiotic repressive effect of *Sly* on genes encoded on the X and Y chromosomes drove their massive amplification in the mouse.

## Introduction

During spermatogenesis, germ cells progress through three phases to become functional sperm: proliferation, meiosis, and spermiogenesis. In the latter phase, haploid germ cells (spermatids) undergo dramatic remodeling and DNA compaction as they differentiate into spermatozoa.

The X and Y chromosomes are transcriptionally silenced during meiosis by a process termed *meiotic sex chromosome inactivation* (MSCI), and postmeiotically, the spermatid X and Y chromosomes remain largely repressed [1]. Nevertheless, there is substantial X and Y gene expression in spermatids, and based on their analysis of X gene expression in spermatids, Mueller and colleagues have argued that gene amplification plays a key role in compensating for postmeiotic sex chromatin repression (PSCR) [2]. Although the chromatin modifications associated with MSCI and PSCR are not the same [1],[3], PSCR is thought to be a downstream consequence of MSCI [4],[5].

In 2005, we reported the surprising finding that deletions of the long arm of the mouse Y (MSYq) lead to the up-regulation of several spermatid-expressed X and Y chromosomal genes [6]; this suggests that one (or more) of the multicopy genes known to be located on MSYq is involved in PSCR. Aside from this, MSYq deficiencies cause sperm head malformations, with severity correlating with the extent of the deficiency and ultimately leading to infertility [7]–[11]. Intriguingly, males with an approximately two-thirds deletion of MSYq (2/3MSYq^−^) are fertile but produce offspring with a sex ratio distortion in favor of females; this has been considered a manifestation of a postmeiotic intragenomic conflict between the sex chromosomes that led to the amplification of sex ratio distorter and suppressor genes [12]–[14].

Our favored candidate for the MSYq factor needed for normal sperm differentiation and a balanced sex ratio has been *Sly*, one of the four multicopy genes identified on the mouse Y long arm [6],[15],[16]. *Sly* encodes a protein that is very highly expressed in round spermatids, and among the proteins with which it interacts are the acrosomal protein DKKL1 and the chromatin modifier and transcriptional coactivator KAT5 (aka TIP60) [17]. More than 70 copies of *Sly* that retain an open reading frame, and 30 copies annotated as “noncoding” are predicted to be present on MSYq (Entrez Gene database from the National Center for Biotechnology Information [NCBI]; http://www.ncbi.nlm.nih.gov/sites/entrez?db=gene) as a result of the amplification of a >500-kb repeat unit encompassing at least two copies of *Sly* [16] (J. Alfoldi and D. C. Page, personal communication). *Sly* expression is consequently reduced in proportion to the extent of MSYq deficiency [15],[17]. Interestingly, the X chromosome carries multiple copies (∼25) of *Slx* (*Sycp3-like, X-linked*) [2], a gene related to *Sly* that encodes a cytoplasmic spermatid-specific protein of unknown function [18]. *Slx* is one of the X-linked genes found to be up-regulated in MSYq-deficient males, and *Slx* and *Sly* have been suggested to be key players in the postmeiotic X-Y genomic conflict [6].

Because *Sly* is present in multiple copies, traditional gene targeting is not an option for investigating its function. In the present study, by using an in vivo RNA interference approach, we have produced male mice with a dramatic reduction in *Sly* expression. The analysis of these mice has enabled us to demonstrate that SLY is a key regulator of sex chromosome gene expression during sperm differentiation.

## Results

### Transgenic Delivery of *Sly*-Specific Short Hairpin RNAs Leads to a Dramatic Reduction in *Sly* RNA and Protein Levels

**Figure 1:**
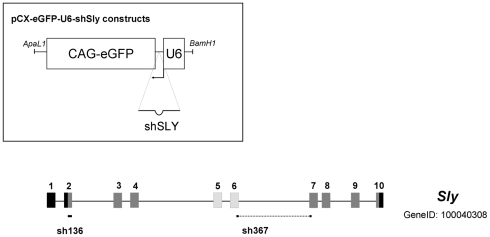
Structure of the *Sly* gene (GeneID 100040308) and of shSLY constructs. The boxes numbered 1 to 10 symbolize exons, with the coding region in grey. Alternatively spliced exons are indicated in hatched grey. The locations of the sequences used to produce the two shSLY constructs (sh136 and sh367) are indicated beneath the schematic of the *Sly* gene. Upper left insert: schematic of the plasmid construct injected to produce shSLY transgenic mice.

The major challenge in the study of MSYq gene functions is the highly repetitive nature of MSYq, which contains coamplified multicopy genes organized in clusters over several megabases [16] (J. Alfoldi and D. C. Page, personal communication). This precludes the use of conventional gene targeting, so we used a transgenic approach to deliver short hairpin RNAs (shRNA) [19] designed to generate *Sly*-specific small interfering RNAs (siRNAs) (Figure 1).

*Sly* short hairpin target sequences were selected to be shared by most *Sly* copies, and to be specific to *Sly*; particular care was taken to avoid sequences that might target related genes such as *Slx*. The selected *Sly* short hairpin sequences (shSLY) were cloned under the control of the U6 promoter [20]. We chose U6, a ubiquitous polymerase III promoter, in order to achieve sufficient expression of shSLY RNA to produce a substantial knock-down of the very abundant *Sly* transcripts. As *Sly* expression is restricted to the testes, no other organs were expected to be affected by its knock-down, and indeed, we did not see any phenotypic changes other than testis-related changes (see below).

**Figure 2:**
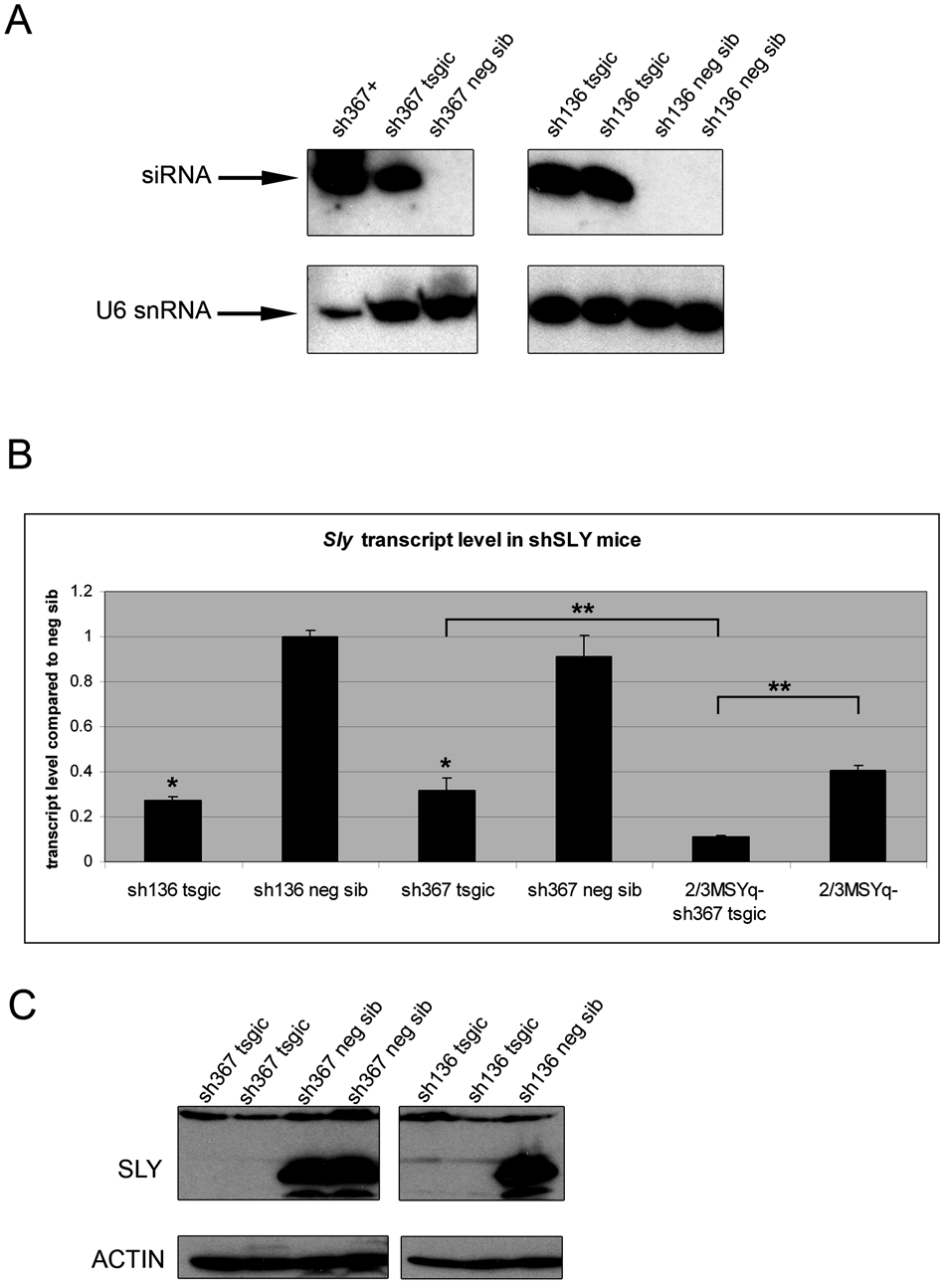
Transgenically delivered shRNAs specific to *Sly* produce a dramatic decrease of *Sly* transcript and protein levels. (A) Northern blot detection of the transgenically delivered small RNAs sh136 and sh367 in testis extracts from shSLY transgenic mice (tsgic) and negative siblings (neg sib). RNA from HEK293 cells transfected with the sh367 transgene was used as a positive control (sh367+). U6snRNA detection was used as loading control. (B) *Sly* transcript levels (using global *Sly* primers F+R1/R2) in shSLY transgenic mice and controls, obtained by real-time PCR. ShSLY transgenic mice with a normal Y^RIII^ chromosome (sh136 or sh367 tsgic) or with an ∼2/3 deletion of MSYq (2/3MSYq^−^ sh367 tsgic) were compared to nontransgenic mice of the same background (sh136/sh367 neg sib or 2/3MSYq^−^). The *y-*axis indicates the level of expression compared to negative siblings (2^ΔΔCt^±standard errors). One asterisk indicates significant difference from the corresponding nontransgenic value (*p*<0.01; *t*-test). Two asterisks indicate significant difference between 2/3MSYq^−^ sh367 and sh367 transgenic mice or between 2/3MSYq^−^ sh367 and 2/3MSYq^−^ mice (*p*<0.005; *t*-test). (C) Western blot detection of SLY protein in testis extract of shSLY transgenic mice (sh136 or sh367 tsgic) and negative sibling (neg sib). Actin detection was used as a loading control.

The efficiency and specificity of shSLY constructs were tested in cell culture by cotransfection experiments (Figure S1). Two shSLY constructs (sh136 and sh367—see Figure 1) were used to produce transgenic mice. High expression of shSLY RNA was associated with a dramatic decrease in *Sly* expression at the transcript and at the protein levels (Figure 2A–2C). Transgenic mice for sh136 and sh367 constructs (hereafter, sh136 and sh367 mice) showed an ∼70% reduction of *Sly* transcripts (Figure 2B), with both known splice variants being affected (unpublished data). SLY protein level was even more dramatically reduced, with no protein being detected by Western blotting (Figure 2C and Figure S2) even after long exposure. This discrepancy suggests that the persisting *Sly* transcripts are not translated, or that they encode a variant SLY protein(s) not detected by our antibody. The sh367 transgene was also introduced into 2/3MSYq^−^ males and *Sly* transcript levels fell further, to 10% of those of normal males (Figure 2B).

Four checks were made for “off-target” effects of the RNA interference. First, the levels of two testis-expressed microRNAs, mir-t3 and mir-t25 [21], were measured and found to be unchanged in testes of shSLY mice (Figure S3A), indicating that the transgenically delivered siRNAs did not affect the expression of naturally expressed small RNAs. Second, since some shRNAs or siRNAs induce an interferon response [22]–[24], the expression level of 2′,5′-oligoadenylate synthetase 1 (*Oas1*) was measured as a marker of an interferon response [22],[24],[25]; this was also unchanged in shSLY mice (Figure S3B). Third, microarray analyses performed on sh367 mice did not detect any significant changes in the expression of known target genes of the interferon pathway (see below). Finally, microarray analyses were performed on juvenile testes (17 d postpartum) to check for potential off-target gene activation before the onset of *Sly* expression. There were no statistically significant differences in gene expression between juvenile sh367 mice and controls (unpublished data).

In view of the substantial and specific knock-down of *Sly* expression in the shSLY mice, we proceeded to analyze their phenotypes in order to assess the extent to which *Sly* depletion mimicked the phenotypic consequences of MSYq deficiencies.

### *Sly* Deficiency Leads to a Marked Increase in Sperm Head Abnormalities and Delayed Sperm Shedding

**Figure 3:**
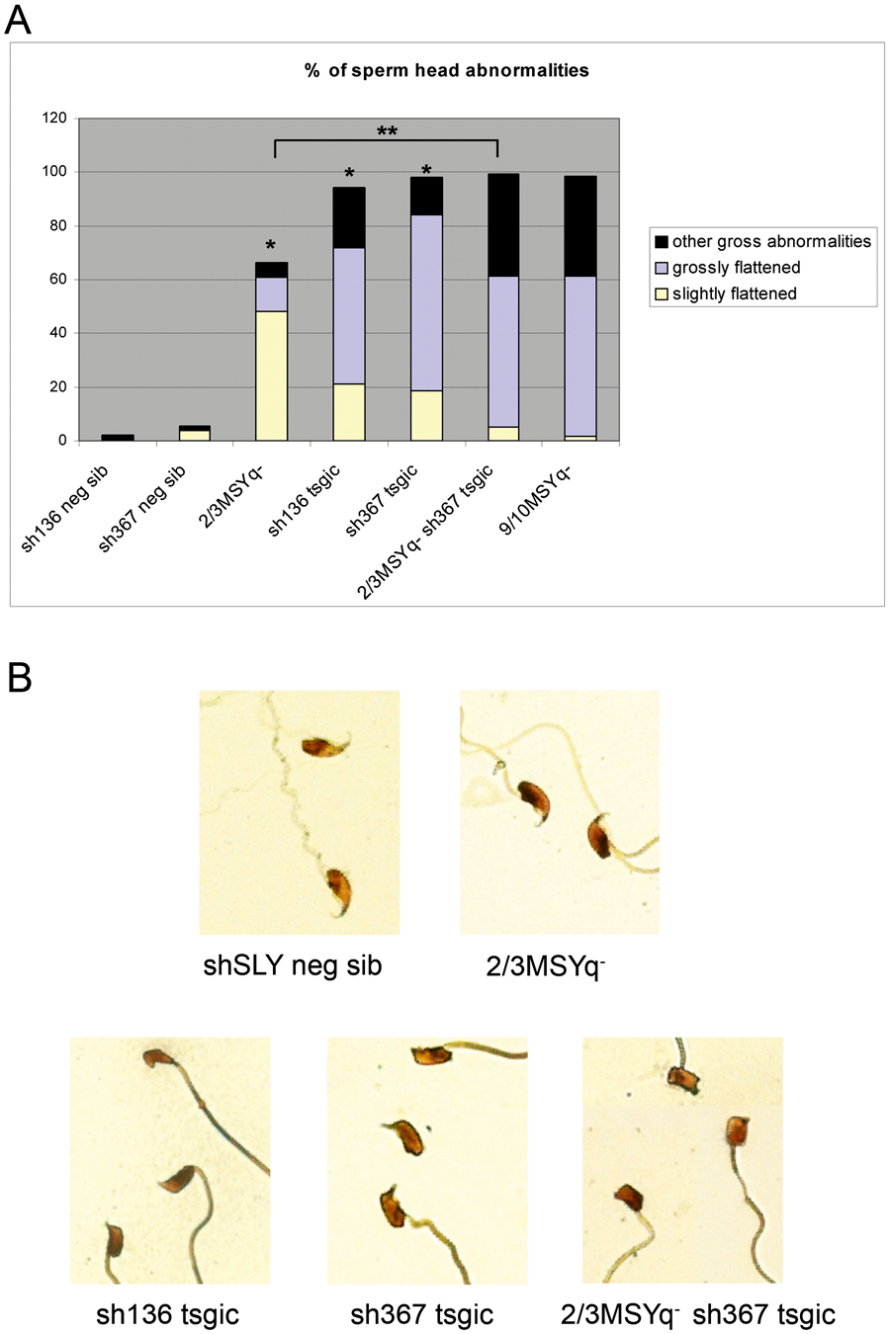
Sperm head abnormalities in *Sly*-deficient mice. (A) Bar graph representing the percentage of sperm head abnormalities in shSLY transgenic mice and controls. ShSLY transgenic mice with a normal Y^RIII^ chromosome (sh136 or sh367 tsgic), or a Y^RIII^ with an ∼2/3 deletion of MSYq (2/3MSYq^−^ sh367 tsgic), were compared to nontransgenic mice of the same background (sh136/sh367 neg sib or 2/3MSYq^−^). Nontransgenic 2/3MSYq^−^ mice were also compared to nontransgenic Y^RIII^ mice. The graph shows the proportion of each category of sperm head abnormalities per genotype. One asterisk indicates significant difference from the corresponding non transgenic Y^RIII^ value (*p*<0.0001; ANOVA). Two asterisks indicate significant difference between 2/3MSYq^−^ sh367 transgenic and 2/3MSYq^−^ mice (*p*<0.0001; ANOVA). (B) Examples of sperm head abnormalities observed in these mice (silver-stained epididymal sperm).

Mice with MSYq deficiencies have an increased incidence of sperm head abnormalities, correlated with the extent of the deletion [7]–[11]. In mice lacking 9/10ths of MSYq (9/10MSYq^−^) or with no MSYq (MSYq^−^), 100% of the sperm are abnormal, and this is thought to be the cause of their sterility [10],[11]. Analysis of epididymal sperm from *Sly*-deficient mice (i.e., shSLY mice from either line) revealed that over 92% of the sperm had head abnormalities, and defects were similar to those observed in mice with MSYq deficiencies (Figure 3A and 3B). The proportion of abnormal sperm heads (grouped into three categories: slightly flattened, grossly flattened, and other gross abnormalities) was very significantly increased (*p*<0.0001) in shSLY mice compared to controls (transgene-negative siblings). In terms of severity, the sperm head abnormalities of shSLY mice from both sh136 and sh367 lines were very similar and fell between those of 2/3MSYq^−^ and 9/10MSYq^−^ mice (Figure 3A). The presence of the sh367 transgene in the context of 2/3MSYq^−^ led to a more severely abnormal sperm phenotype than that seen in 2/3MSYq^−^ males or in shSLY males with a normal Y^RIII^ chromosome (Figure 3A and 3B, Figure S4).

Another spermiogenic defect described for 9/10MSYq^−^ males [11] and seen in MSYq^−^ males (unpublished data) is a delay in sperm shedding. This is also the case for shSLY mice (unpublished data).

Together these results show that the key spermiogenic defects observed in MSYq^−^ mice are also seen in shSLY mice, thus demonstrating that *Sly* deficiency is the underlying cause.

### *Sly* Deficiency Leads to Impaired Sperm Function with Consequent Reduced Fertility

Previous studies have shown that 9/10MSYq^−^ and MSYq^−^ males are sterile [10],[11], whereas males carrying less extensive deletions of MSYq (such as 2/3MSYq^−^ and B10.BR-Ydel) are fertile, but their sperm have markedly reduced in vitro fertilizing ability [12],[26]–[28]. We therefore checked for impaired fertility in shSLY males, initially focusing our study on sh367 males since the phenotypes of both lines were similar. As is the case for 9/10MSYq^−^ and 2/3 MSYq^−^ males, testis weights for the shSLY males did not differ from controls; sperm numbers were reduced but within the fertile range (Table S1). However, when mated for a period of 7 mo, sh367 mice had markedly fewer offspring and litters when compared to transgene-negative siblings (Table 1). The in vitro fertilizing ability of epididymal sperm samples was also dramatically reduced relative to controls with only one of 662 eggs developing to the two-cell stage (Table 1), and the quality of sperm motility appeared to be impaired as shown by the increase in the proportion of non-progressively motile sperm (Table S1). A reduced quality of motility has recently been reported for B10.BR-Ydel males [29]. Overall, the fertility defects were intermediate in severity between those of 2/3MSYq^−^ and 9/10MSYq^−^ mice, as was the case for the sperm head abnormalities. Surprisingly, two sh136 and sh367 transgenic males that were obtained early in the backcrosses were exceptionally fertile, whereas *Sly*-deficient males obtained in subsequent generations are all markedly subfertile or sterile (see Table S2).

**Table 1:** In vitro and in vivo analysis of the fertilizing ability of *Sly*-deficient mice compared to controls.

Type	Category	sh367 tsgic	Controls
**IVF**	% of two-cell embryos obtained from F1 oocytes	0** (1/395)	17 (54/316)
	% of two-cell embryos obtained from MF1 oocytes	0** (0/267)	22 (47/217)
**Breeding**	Number of litters per male	2.5*	9
	Total number of offspring per male	11*	70.5
	Average number of offspring per litter	4.83	7.83

Statistical significance of difference with respect to corresponding control:

*: *p*≤0.005.

**: *p*≤0.001.

Because of the poor fertility of *Sly*-deficient males, offspring sex ratio data are very slow to accumulate. After pooling data for all matings involving sh136 and sh367 males and for nontransgenic males arising in the same breeding program, 55.5% (79 of 142) of the offspring of the transgenics are female and 47.8% (176 of 368) of the offspring from nontransgenics are female. The sex ratio distortion in favor of females (7.7%) approaches significance at the 0.05 level (*p*-value = 0.0569). However, a major caveat is the fact that a large proportion of the data come from just two males (Table S2). It may be necessary to produce an shSLY line with an intermediate knock-down to establish whether or not *Sly* deficiency contributes to the sex ratio distortion observed in 2/3MSYq^−^ mice.

### *Sly* Deficiency Leads to a Global Derepression of X-Y Genes following Meiosis

**Figure 4:**
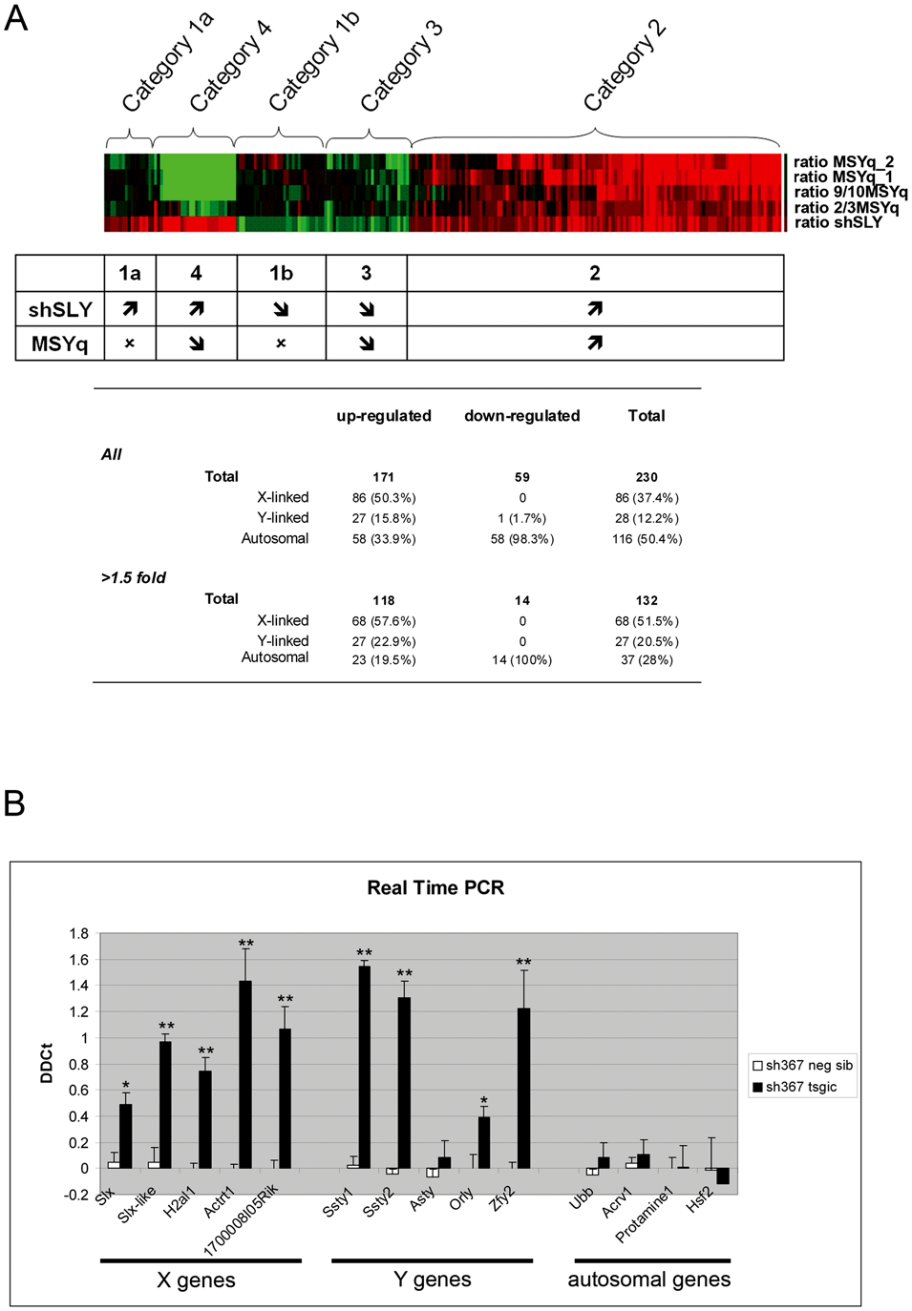
X-Y gene up-regulation in *Sly*-deficient mice. (A) Results from the microarray analyses performed on testes of *Sly*-deficient mice (shSLY 367) and MSYq-deficient mice (2/3MSYq^−^, 9/10MSYq^−^, and MSYq^−^). MSYq^−^ samples came from 1- or 2-mo-old mice (respectively, MSYq_1 and MSYq_2 on the heat map). Differentially expressed genes were grouped into five categories based on their expression ratios across all genotypes. The red and green colors of the heat map represent gene up-regulation and down-regulation, respectively. Categories 1a and 1b represent genes found up-regulated (up arrows) or down-regulated (down arrows) in sh367 mice, without consistent changes (x) in MSYq^−^ models. Genes in category 2 were up-regulated in sh367 and MSYq^−^ mice with some dose-dependency with the extent of MSYq deletion. Genes in category 3 were down-regulated in sh367 and MSYq^−^ mice with some dose-dependency with the extent of MSYq deletion. Finally, category 4 groups genes found to be up-regulated in sh367 mice and down-regulated in MSYq^−^ mice, with a very strong dose-dependency with the extent of Y deletion. The table indicates the number and the chromosomal distribution of the genes that were found differentially expressed in sh367 mice compared to nontransgenic siblings. (B) Analysis of gene expression in testis of *Sly*-deficient mice (sh367 tsgic) and controls (neg sib) by real-time PCR. Values plotted are ΔΔCt±standard errors. One or two asterisks indicate significant difference from the control value (respectively, *p*<0.05 or *p*<0.005; *t*-test).

In our initial gene expression study of MSYq-deficient mice, 18 sex chromosome genes (of which 16 were X- and two Y-linked) were found up-regulated [6]. We decided to investigate the consequences of *Sly* deficiency on gene expression in our new mouse model, and to reexamine gene expression in MSYq-deficient males, using a more exhaustive array. Microarray analyses were performed on adult testes of sh367 mice, sibling controls, 2/3MSYq^−^, 9/10MSYq^−^, and MSYq^−^ mice (see Figure S5). We found 230 differentially expressed genes that were grouped into five categories based on their expression ratios across all genotypes (Figure 4A). The largest category (127 genes) comprises genes that are up-regulated in sh367 mice and in mice with MSYq deficiencies (category 2); 67.7% (86/127) of these up-regulated genes are X-linked, increasing to 81% (68/84) of those up-regulated at least 1.5-fold (Figure S5). Many of these X-linked genes are present in multiple copies, such as *Slx*, *Cypt*, *Asb*, *Ssxb*, and *Rhox*; of these, *Slx*, *Cypt*, *Ssxb*, *Rhox3*, and *Rhox11* are specifically expressed in postmeiotic cells (Table 2). Several of the up-regulated single-copy X-linked genes are also known to be involved in the differentiation of postmeiotic germ cells (i.e., spermatids) (Table 2). Indeed, *Actrt1* and *Spaca5* are components, respectively, of the perinuclear theca and the acrosome, two highly differentiated structures of the sperm head [30],[31]. X-linked genes encoding variants of histone H2A (*LOC100045423*, a copy of *H2al1*, and *LOC100046339*, which is closely related to *H2A.Bbd*) were also derepressed in shSLY and MSYq-deficient mice (cf. Table 2).

**Table 2:** Examples of transcripts found up-regulated in *Sly*- and MSYq-deficient mice (category 2) and their chromosomal location.

Type	Family	Additional Information [Reference]	Gene	Accession	Map^a^
**Multicopy**	***Asb***		*Asb9*	NM_027027	X
			*Asb12*	NM_080858	X
	***Cypt***	Postmeiotic-specific expression [33]	*Cypt2*	NM_173436	X
			*Cypt3*	NM_173367	X
			*Cypt6*	NM_025738	X
			*Cypt9*	NM_001039942	U
			*Cypt10*	NM_001039944	U
	***Hist1h3***		*Hist1h3a*	NM_013550	13
			*Hist1h3e*	NM_178205	13
	***Hist1h4***		*Hist1h4a*	NM_178192	13
			*Hist1h4c*	NM_178208	13
			*Hist1h4f*	NM_175655	13
			*Hist1h4i*	NM_175656	13
	***H2al1***	Postmeiotic-specific expression [45],[46]	*LOC100045423*	XM_001472536	X
	***Rhox***	Postmeiotic-specific expression [49]	*Rhox3a*	NM_194063	X
			*Rhox3h*	XM_885355	X
			*Rhox11*	NM_198598	X
	***Slx***	Postmeiotic-specific expression [18]	*LOC666096*	XM_981599	X
			*MGC118210*	NM_001025607	X
			*LOC100039120*	XM_001472448	X
			*EG546282*	NM_001081657	X
			*LOC382213*	(discontinued)	X
	***Speer***	Postmeiotic-specific expression	*Speer4a*	NM_029376	5
			*Speer4c*	XM_890800	5
			*LOC664837*	XM_973329	5
			*LOC100038949*	XM_001471959	5
			*LOC100039045*	XR_030559	5
	***Ssxb***	Postmeiotic-specific expression [2]	*Ssxb5*	NM_199319	X
			*Ssxb10*	NM_199064	X
			*LOC631002*	NM_001081565	X
**Single copy**		Perinuclear theca protein [30]	*Actrt1*	NM_028514	X
		Involved in sperm motility [50]	*Akap14*	NM_001033785	X
		Acrosomal protein [31]	*Spaca5*	XM_001006691	X
		Related to *H2A Bbd*	*LOC100046339*	XM_001472670	X
		Related to *TCP11*	*1700008I05Rik*	NM_027952	X

a The two unmapped genes (U) are most likely located on the X chromosome.

The array also shows up-regulation in sh367 mice of 27 Y-linked gene loci, almost exclusively representing the multicopy genes *Ssty1* and *Ssty2* (category 4). *Ssty1* and *Ssty2* are spermatid-specific MSYq-encoded genes [32] and are consequently reduced in mice with MSYq deletions (Figure S5). Overall, more than 65% of the genes up-regulated in shSLY mice, and >80% of the ones that are highly up-regulated (>1.5-fold increase), are located on the sex chromosomes (Figure 4A). No X or Y genes (except one pseudogene provisionally mapped to the Y) were found to be down-regulated.

The microarray results for a number of genes were confirmed by real-time PCR (Figure 4B). Spermatid-specific X- and Y-linked multicopy genes, such as *Slx*, *Slx-like*, *H2al1*, *Ssty1*, and *Ssty2* are all markedly derepressed in shSLY mice, and this is also the case for the spermatid-specific single-copy genes *Actrt1* and *1700008I05Rik* (an X-linked homolog of the t-complex gene *Tcp11*). Of two other MSYq-encoded spermatid-expressed genes that were not on the array, *Asty* was not significantly up-regulated, and *Orly* was not as dramatically up-regulated as *Ssty1* and *Ssty2*. *Zfy2*, another gene predominantly expressed in spermatids [33], was found markedly up-regulated by real-time PCR, even though not picked up in our array analysis. *Zfy2* is encoded by the Y chromosome short arm, and its up-regulation shows that the derepression of the Y is not restricted to its long arm. Levels of expression of autosomal genes, including the spermatid-specific *Acrv1* and *Protamine1* genes, are not significantly changed, in agreement with the sex-linked gene bias identified in the microarray (Figure 4B). Similar results were obtained for sh136 transgenic mice (unpublished data).

A further microarray analysis was performed on purified round spermatids from sh367 transgenic mice, sibling controls, and 2/3MSYq^−^ mice. The vast majority of the X and Y genes found up-regulated before were also found significantly up-regulated in the new comparison (109 of 113, Figure S6). In addition, this new dataset identified a greater number of up-regulated X-linked genes (126 vs. 68 genes, >1.5-fold increase; see Figure S6). The identification of a greater number of genes in the new dataset is probably due to the increase in sensitivity when the analysis is restricted to the cell type in which up-regulation occurs. Additional Y-encoded up-regulated transcripts were also identified, such as *Orly*, *Rbm31y*, and *H2al2y* (a Y-encoded histone H2A spermatid specific variant).

This demonstrates that the derepression of sex chromosome genes occurs in spermatids and also provides a control for differences in the cellular composition between *Sly-*deficient, MSYq-deficient, and control testes.

**Figure 5:**
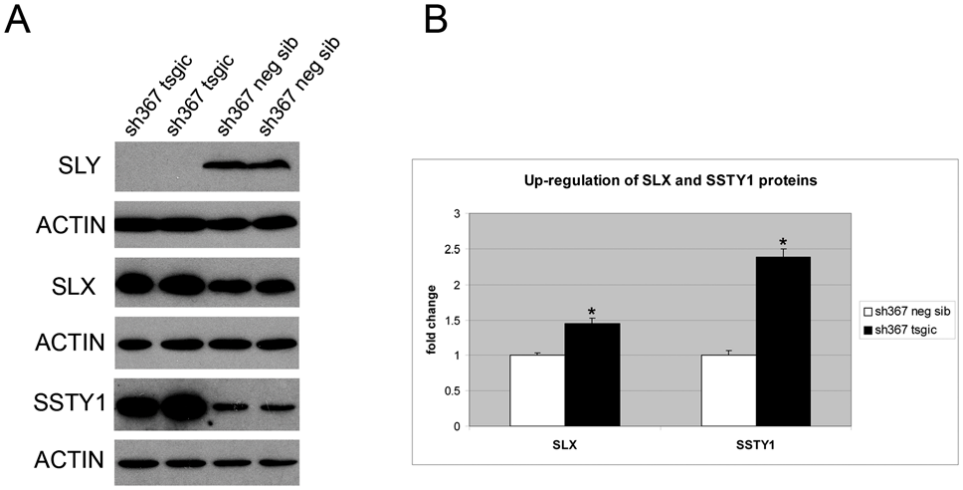
X-Y gene up-regulation in *Sly*-deficient mice, at the protein level. (A) Western blot detections of the spermatid-specific proteins SLX, SSTY1, and SLY in testes of *Sly*-deficient mice (sh367 tsgic) and controls (neg sib). (B) Levels of expression were quantified using ImageJ software and normalized with respect to actin signal. SLX and SSTY1 proteins, encoded, respectively, by X and Y genes found to be up-regulated at the transcript level, are significantly increased in testis of sh367 transgenic mice compared to control. Similar results were obtained for sh136 transgenic mice (unpublished data). Statistical significance with respect to corresponding control: *p≤0.0005 (*t*-test).

The up-regulation of sex-chromosome spermatid genes observed at the transcript level is also detected at the protein level, as shown for SLX and SSTY1 proteins (Figure 5A and 5B and Figure S2). SLX immunostaining of testis sections confirmed that the derepression is restricted to spermatids (Figure S7).

All these data point to a global derepression of postmeiotic sex chromatin (PMSC) following *Sly* deficiency; the X and Y genes that are up-regulated are those already expressed in spermatids. Thus, it is clear that SLY has a key role in PMSC repression.

### *Sly* Deficiency Leads to Reduced Heterochromatin Marks on the PMSC

Several studies have demonstrated that the PMSC of X and Y spermatids is enriched in histone modifications known to be associated with transcriptional repression, such as hypermethylation of lysine 9 of histone H3 (H3K9) [3]–[5],[34],[35]. The heterochromatin proteins CBX1 and CBX3 (aka HP1β and HP1γ) also accumulate on PMSC [4],[5],[35]. As shown in other contexts, the heterochromatin proteins are recruited via binding to methylated H3K9 [36],[37] and mediate gene repression [38],[39].

**Figure 6:**
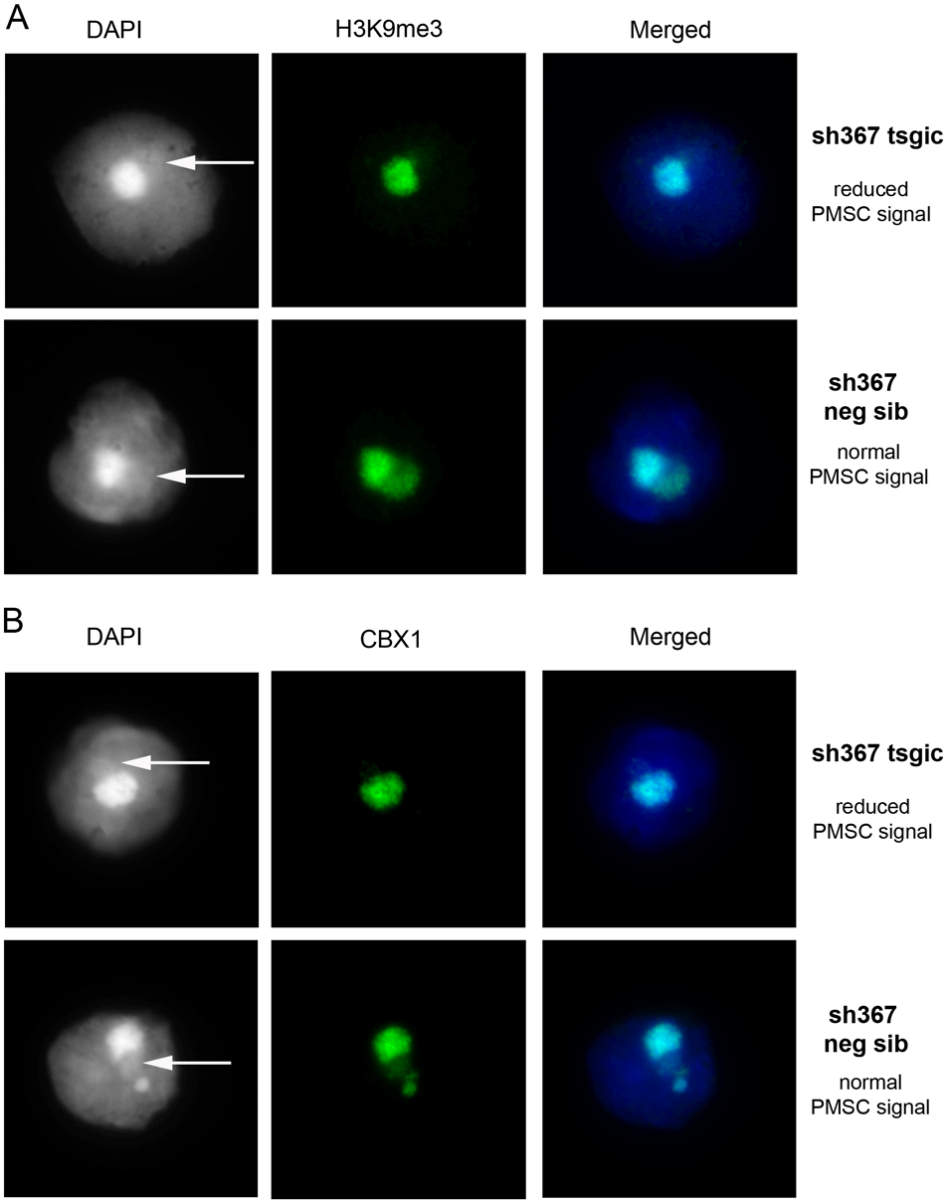
Decreased H3K9me3 and CBX1 staining on the sex chromatin of *Sly*-deficient spermatids. Detection of (A) H3K9me3 and (B) CBX1 by immunofluorescence in surface-spread round spermatids. DAPI (white, or blue in the merged picture) was used to stain nuclei. The round DAPI-dense structure is the chromocenter. The less DAPI-dense structure at the periphery of the chromocenter is the postmeiotic sex chromatin (PMSC) and is indicated by an arrow. Pictures were taken using the same image capture parameters.

In view of our microarray results implying global PMSC derepression in *Sly*-deficient spermatids, we decided to examine these repressive chromatin marks in our mouse model. The analysis of shSLY mice revealed a significant (*p*<0.005) decrease of trimethylated H3K9 (H3K9me3) staining on PMSC as compared with the chromocenter (85% of spermatids with less staining vs. 46.5% in control mice). Similarly, CBX1 accumulation on PMSC was significantly (*p*<0.05) reduced in mutant mice relative to the chromocenter (82% of spermatids with less staining vs. 57% in control mice) (Figure 6 and Figure S8). A recent study shows a comparable reduction of PMSC-associated H3K9me3 and CBX1 staining in MSYq-deficient males [40].

These observations imply that the repressive effect of SLY on sex chromosome gene expression in spermatids is due to a global effect on PMSC via ubiquitous mediators of heterochromatinization/transcriptional silencing.

Despite the fact that SLY has been shown to interact with the histone acetyl transferase KAT5 [17], so far no obvious change of histone acetylation was detected in *Sly*-deficient round spermatids (unpublished data). KAT5 is highly expressed in spermatocytes but poorly expressed in spermatids ([41] and unpublished data), and it is possible that the effect of SLY on KAT5 function is too subtle to be observed with our current tools.

### SLY Protein Colocalizes with the PMSC

**Figure 7:**
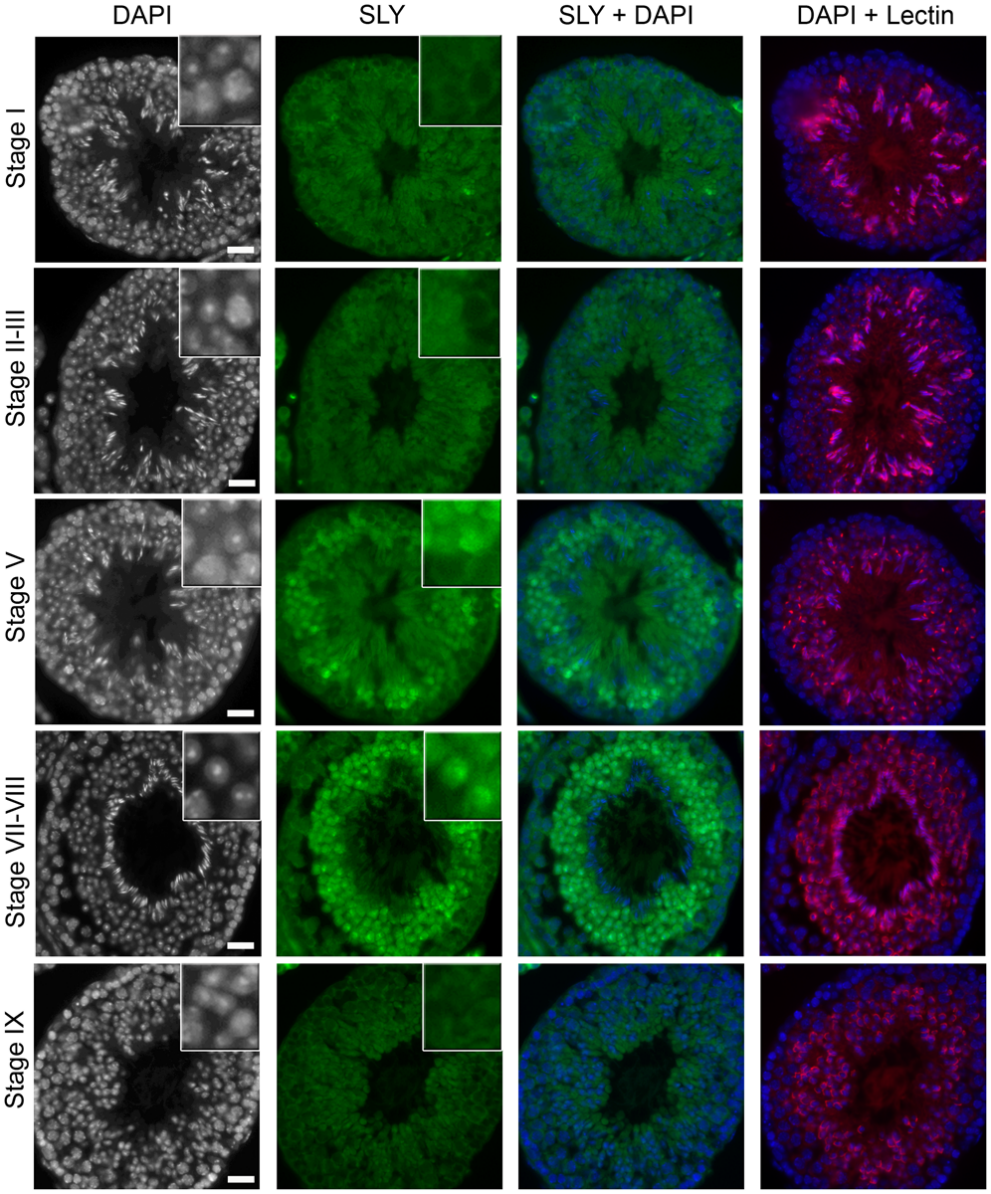
SLY protein is located in the spermatid nuclei in a stage-specific manner. Detection of SLY protein (green) by immunofluorescence in wild-type testis sections. DAPI (white, or blue in the merged picture) was used to stain nuclei, and lectin-PNA (red) was used to stain acrosomes in order to determine tubule stage. The inset in the upper-right corner represents a 3.4× magnification. Pictures were taken using the same image capture parameters. Scale bars indicate 20 µm. See Figure S9 for a complete panel.

**Figure 8:**
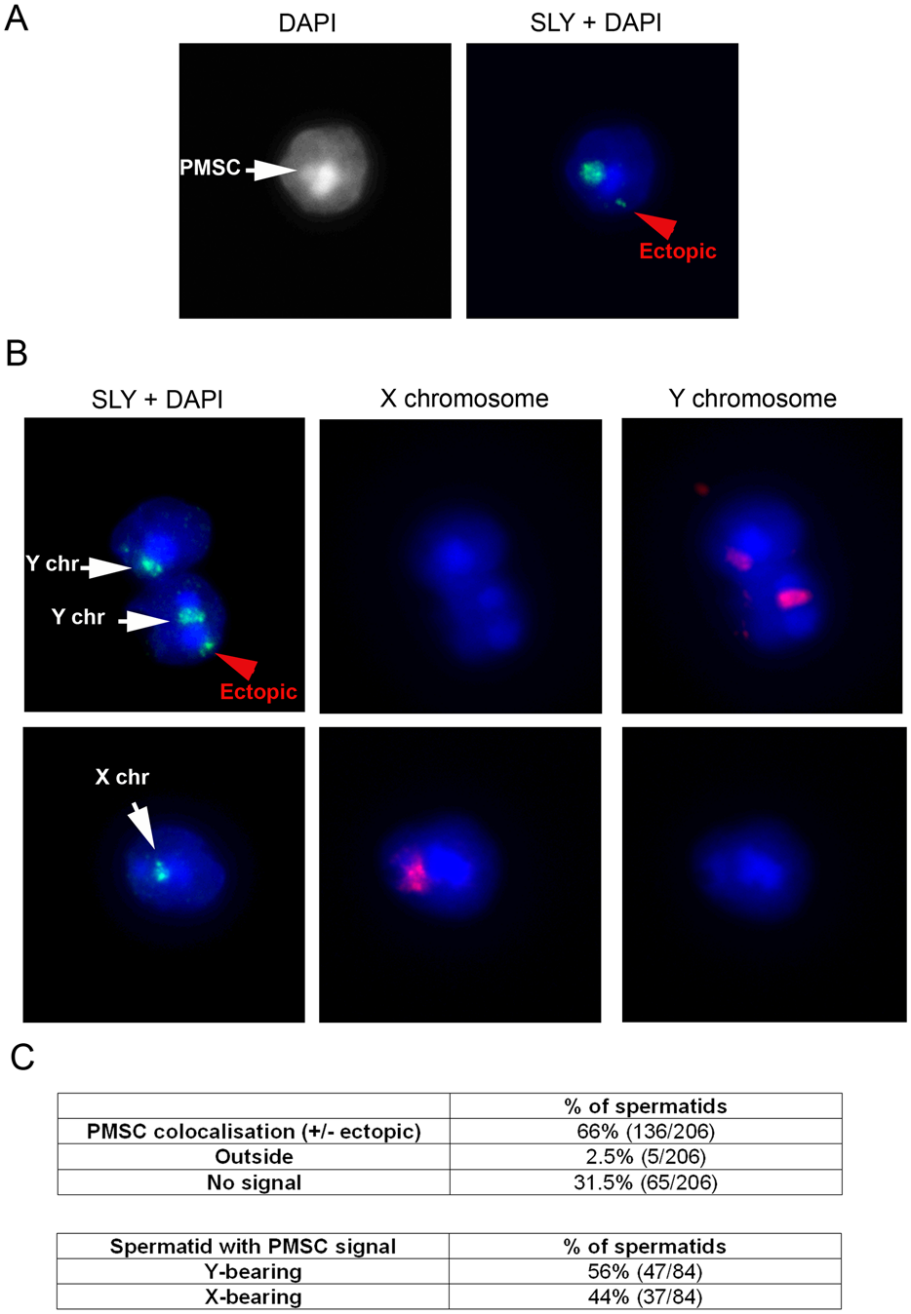
SLY protein colocalizes with the sex chromatin in round spermatids. (A) and (B) Detection of SLY protein (green) by immunofluorescence in surface-spread spermatids. DAPI (white or blue) was used to stain nuclei. SLY protein colocalizes with the PMSC (white arrow) in round spermatids. A fainter signal is sometimes observed outside PMSC (ectopic, red arrowhead). X and Y chromosome painting shows that SLY colocalizes with either sex chromosome in spermatids. See Figure S10 for controls. (C) The tables indicate the percentage of spermatids with SLY PMSC staining, and the percentage of X-bearing or Y-bearing spermatids with SLY PMSC staining.

The SLY protein is related to SYCP3 and XLR, two nuclear proteins thought to associate with chromatin via their conserved COR1 domain (NCBI Conserved Domains Database; http://www.ncbi.nlm.nih.gov/Structure/cdd/cdd.shtml) [15]. Based on cytoplasmic/nuclear protein extracts, SLY protein is predominantly located in the cytoplasm of round and early-elongating spermatids, but a significant fraction is nevertheless observed in the nucleus [17]. However, the nuclear localization has not been documented by immunostaining. Using modified immunohistochemistry protocols, we have now been able to observe SLY protein in the nuclei of spermatids from stage II–III until early stage IX, with the intensity of the signal increasing through spermatid development. SLY nuclear staining is then excluded from the nuclei at the onset of spermatid elongation (from stage IX, see Figure 7 and Figure S9). SLY nuclear localization is consequently specific to round spermatids (probably excluding stage I round spermatids in which SLY nuclear staining was not detectable above background). At higher magnification, SLY seemed to localize to a DAPI-dense subnuclear structure that could be the PMSC (Figure S9C). This was confirmed in spread spermatids in which SLY clearly colocalized with either the X or the Y PMSC in 66% of round spermatids (Figure 8 and Figure S10). In addition to PMSC colocalization, SLY protein was sometimes observed outside PMSC (ectopic, Figure 8); the reason for this non-PMSC localization remains to be determined. The 31.5% of spermatid nuclei without an SLY signal may be accounted for by the absence of nuclear SLY in early-stage spermatids.

These data strongly suggest that SLY induces gene repression via direct interaction with the PMSC or with PMSC protein partners such as histone-modifying enzymes.

### *Sly* Deficiency Does Not Alter DKKL1 Level or Localization

In a previous study, we observed that SLY interacts with the acrosomal protein DKKL1 [17]; the severe defects of *Sly*-deficient sperm could be a consequence of the disturbance of this interaction. However, DKKL1 intracellular localization was unchanged in *Sly*-deficient testes (Figure S11), and the global level of DKKL1 protein in purified spermatids was also unaffected (Figure S2).

### *Sly* Deficiency Leads to Deregulation of Several Autosomal Genes

In addition to the massive up-regulation of sex chromosome genes expressed in spermatids, some autosomal genes were identified as up-regulated in *Sly*- and MSYq-deficient mice (see Figure 4A; and category 2, Figure S5). Intriguingly, these included five members of the multicopy *Speer* gene family and several autosomal genes encoding variants of histones H3 and H4 (Table 2). Genes that were down-regulated in shSLY and in MSYq-deficient mice (category 3) were exclusively autosomal (aside from a provisionally Y-linked pseudogene). These down-regulated genes include *Chaf1b*, a chromatin assembly factor (Figure S5). The microarray analysis on purified round spermatids confirmed the change of expression for the majority of these autosomal genes (Figure S6).

Other autosomal genes were found to be up-regulated or down-regulated in shSLY mice, but not in MSYq^−^ mice (category 1a and 1b, Figure S5). This could be due to off-target effects of shSLY transgene expression, but no particular pattern/pathway was apparent. Genes of the interferon pathway, previously reported to be activated by some shRNAs [22]–[24], were not up-regulated in shSLY mice, supporting the evidence from the microarray analyses performed before the onset of *Sly* expression that there are no off-target effects. Alternatively, the changes in autosomal gene expression that are specific to shSLY mice could be a consequence of the up-regulation of other MSYq genes such as *Ssty1* and *Ssty2* transcripts; in MSYq-deficient models, all MSYq genes show reduced expression.

## Discussion

Using transgenically delivered shRNAs, we have successfully disrupted the function of *Sly*, an MSYq-encoded multicopy gene. This demonstrated first, that SLY has a critical role in PSCR, and second, that *Sly* deficiency is the major underlying cause of the spectrum of anomalies identified 17 y ago in MSYq-deficient males.

### SLY Globally Represses the X and the Y Chromosomes after Meiosis

Our previous study suggesting that MSYq encodes information required for the repression of PMSC identified 18 up-regulated sex-linked genes in testes of MSYq-deficient mice: 16 from the X chromosome and two from the Y chromosome short arm [6]; many of these genes were exclusively expressed in spermatids. Here, our more extensive analysis of MSYq-deficient mice together with *Sly*-deficient mice identified 113 up-regulated sex-linked genes: 86 X-linked and 27 Y-linked. The latter mice served to establish that the up-regulation is due to *Sly* deficiency and that this up-regulation includes multicopy MSYq genes such as *Ssty1* and *Ssty2*. A further microarray confirmed the up-regulation in purified spermatids. SLY is consequently the MSYq factor required for PSCR in the mouse, and the generality of the sex-linked gene repression demonstrates that SLY acts to globally repress the PMSC.

### The Repressive Effect of SLY Is Mediated by Maintaining Heterochromatin Protein Enrichment in the PMSC

How does SLY mediate this global repression of the PMSC in X and Y spermatids? Importantly, we have shown that SLY is nuclear from stage II to IX and localizes to the PMSC of X and Y spermatids. This localization may involve the SLY COR1 domain, which is proposed to mediate association with chromatin (NCBI Conserved Domains Database) [15]. The localization to X-bearing spermatids will have been facilitated by the sharing of gene products via intercellular bridges [42].

In the male germline, the X and Y chromosomes are initially transcriptionally inactivated at the beginning of pachytene (meiotic sex chromosome inactivation [MSCI]); this inactivation is triggered by the phosphorylation of the histone H2AX [1]. During the transition from MSCI to PSCR, there are changes in nucleosomal histones, in epigenetic histone marks, and in the recruitment of heterochromatin proteins; these chromatin features are lost from the PMSC during stage XI [3]–[5],[34],[35],[43]. This loss at stage XI is unsurprising since it is the stage when the replacement of histones with protamines is initiated.

Of importance in the current context is the recruitment of the heterochromatin protein CBX1 during diplotene, coincident with the loss of H2AX phosphorylation, that is presumed to be responsible for sex chromosome repression following the shutdown of MSCI [4],[5],[43],[44]. It is thus significant that we have found that SLY subsequently plays a role in maintaining CBX1 enrichment in the PMSC, and thus, in maintaining a substantial degree of transcriptional repression. Hypermethylated H3K9 is known to be a platform for CBX1 recruitment [36],[37], and in view of the changes in nucleosomal histones during the MSCI-PSCR transition [3], maintenance of CBX1 enrichment is likely to require continuing H3K9 methylation as the new histones are introduced. It is therefore noteworthy that SLY is also involved in maintaining H3K9 trimethylation.

In our microarray screening, several autosomal and sex-linked genes coding for histones H2, H3, and H4 variants (including recently identified spermatid-specific H2A variants, *H2al1* and *H2al2y* [45],[46]) were found up-regulated when *Sly* expression is reduced. Conversely, *Chaf1b*, which encodes a chromatin assembly factor, appears down-regulated. All these changes could contribute to the derepression of sex chromatin. The importance of histone H3 (and particularly of variant H3.3) in germline function has been recently described in the mouse and *Drosophila* [3],[47].

### The Sperm Defects of MSYq- and *Sly*-Deficient Mice Are a Consequence of the Derepression of Sex-Linked Spermiogenic Genes

What is the molecular basis for the link between *Sly* deficiency and the spermiogenic defects in MSYq-deficient mice? SLY interacts with the acrosomal protein DKKL1 [17], but our study shows that DKKL1 level and pattern of expression are not noticeably affected by *Sly* deficiency. However, the reduction/absence of SLY leads to a dramatic up-regulation of many X and Y genes in spermatids. This up-regulation is almost certainly not benign, and we propose this to be the major contributing factor to the spermiogenic defects of *Sly-* and MSYq-deficient mice.

*Actrt1* [30], *Spaca5* [31], and *Cypt* [33], which are up-regulated in *Sly*-deficient spermatids, encode proteins of the perinuclear theca and the acrosome, two specific structures of the sperm head, and thus are candidates for contributing to sperm head defects. Candidates for effects on sperm function include *1700008I05Rik*, an X-linked homolog of *Tcp11*, which codes for a receptor of a fertilization promoting peptide thought to promote sperm capacitation/function [48]; *Rhox3a*, *Rhox3h*, and *Rhox11*, related to *Rhox5*, which has been implicated in sperm production and motility [49]; and the A-kinase anchoring protein *Akap14*, which is predicted to regulate flagellum function, and consequently, sperm motility [50]. Future work on these many candidate genes will be required to determine their involvement in MSYq-deficient spermiogenesis phenotypes.

### Is *Sly* Deficiency Solely Responsible for the Spermiogenesis Defects of MSYq-Deficient Mice?

If the sperm abnormalities in MSYq-deficient mice are solely a consequence of *Sly* deficiency, then there should be a correlation between the extent of *Sly* reduction and the severity of the sperm defects. The relevant genotypes in order of decreasing transcript levels (given as percentage of control) are: 2/3MSYq^−^ (40%), sh367 (30%), sh367 2/3MSYq^−^ (10%), and 9/10MSYq^−^ (<1%) [6]; this is the same order as that for increasing severity of sperm head defects. However, the latter three genotypes were indistinguishable with respect to the expression of SLY protein, since none could be detected by Western analysis. From sequence data for the remaining transcripts, it appears that the majority encode variant (but presumably functional) SLY proteins that are unlikely to be detected by our antibody, thus providing an explanation for the seeming discrepancy between RNA and protein levels. Nevertheless, it is important to bear in mind that 9/10MSYq^−^ mice differ from shSLY mice in that the former are deficient in other MSYq-encoded transcripts (i.e., *Ssty1*, *Ssty2*, *Asty*, and *Orly*), which could contribute to the severity of their sperm defects.

### X-Y Gene Amplification in Mouse Is a Consequence of the Enhancement of PSCR Resulting from *Sly* Amplification

There is now substantial data documenting that many sex-linked spermatid-expressed genes in the mouse are highly amplified [2],[33],[46], with MSYq genes being especially highly amplified [15],[16] (J. Alfoldi and D. C. Page, personal communication). There are currently two hypotheses that seek to explain this amplification: first, that it is a response to PSCR enabling sufficient expression of some X and Y genes with critical postmeiotic functions [2], and second, that it is driven by a genomic conflict involving postmeiotic competition between X- and Y-encoded gene products that affect sex ratio [13],[14].

Given the variety of genes involved, the PSCR-amplification hypothesis is attractive since it explains why so many different genes have become simultaneously amplified in the mouse: it seems unlikely that they could all affect sex ratio. However, a challenge for the hypothesis is to explain why the same degree of amplification is not seen on the Y chromosome of other species (J. Alfoldi and D. C. Page, personal communication). This can be resolved by our finding that one of the amplified mouse-specific genes, *Sly*, regulates PSCR (our present data). We therefore propose that the mouse-specific expansion of sex-linked spermatid-expressed gene copy number is a downstream consequence of the enhancement of PSCR that accompanied *Sly* amplification.

**Figure 9:**
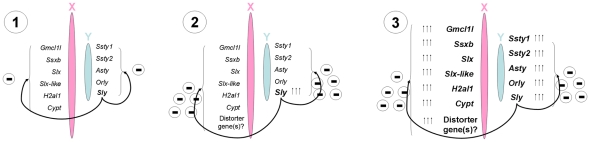
Model of the involvement of *Sly* in the amplification of postmeiotically expressed genes on the mouse X and Y chromosomes. (1) *Sly* functions in PSCR, having a repressive effect on sex chromosome-linked spermatid genes such as *Gmcl1l*, *Ssxb*, *Slx*, *Slx-like*, *H2al1*, and *Cypt* on the X and *Ssty1*, *Ssty2*, *Asty*, *Orly*, and *Sly* itself, on the Y. (2) *Sly* becomes amplified in copy number, possibly in response to the presence of a sex ratio distorter on the X. Increasing expression of *Sly* leads to stronger repression of other sex chromosome-linked spermatid genes via PSCR. (3) Copy number amplification of multiple sex-linked spermatid genes to maintain their expression in the presence of increasing PSCR.

So what drove *Sly* amplification? The straightforward answer would be that this was necessary in order to maintain *Sly* function in the face of the enhancement of PSCR, but this creates the paradox that *Sly* has become amplified in order to escape its own repressive effects. This in turn implies that the enhancement of PSCR must also have been of selective advantage; otherwise, this function of *Sly* would have been lost. One possibility is that the enhancement of PSCR was a weapon in a postmeiotic genomic conflict, where one or more of the genes on the X chromosome acts to distort the sex ratio in favor of females, whereas *Sly* acts via PSCR to repress the distorter gene(s) and restore a normal sex ratio. The fact that 2/3MSYq^−^ mice have a sex ratio distortion in favor of females is strong evidence that MSYq does encode a factor or factors that are suppressing sex ratio distortion. For shSLY mice, we observed a mild sex ratio skew of borderline significance. It may be possible to create further shSLY lines with a milder phenotype more comparable to that of 2/3MSYq^−^ mice to enable us to obtain more extensive breeding data than that obtained with the severely subfertile mice in the present study. Our proposals concerning the role of *Sly* in driving sex-linked spermatid-expressed gene amplification are summarized in Figure 9.

In conclusion, SLY has a predominant role in postmeiotic sex chromatin repression, as it is required for the maintenance of the heterochromatin protein CBX1 on PMSC. *Sly* deficiency recapitulates almost all of the phenotypes observed in mice with MSYq deletions. Thus, *Sly* encodes the spermiogenesis factor identified 17 y ago on the Y long arm [10]. Future studies of the many genes that we found differentially expressed in shSLY mice will help in understanding the direct cause(s) of the multiple spermiogenesis defects observed in *Sly*- and MSYq-deficient mice.

Here, we have used transgenic delivery of siRNAs to disrupt the function of a multicopy Y gene, and the same approach could be used for multicopy genes on other chromosomes, for example, *Slx*, a gene related to *Sly*. Furthermore, despite numerous attempts in several laboratories, no one has reported the successful disruption of the function of a single-copy Y gene using traditional gene targeting strategies [51]; transgenic delivery of Y gene–specific siRNAs should be an effective alternative.

## Materials and Methods

### Plasmid Construction, and Generation and Breeding of Transgenic Mice

To generate the U6shSLY constructs, we used a PCR-based approach similar to that described in Harper et al., 2005, using primers designed to generate the shSLY sequences [20] (cf. Table S3). The PCR products were cloned into the pCR2.1 vector and sequenced (TOPO TA Cloning, Invitrogen). The U6shSLY cassettes were then subcloned into pCX-eGFP plasmid [52].

Prior to injection, the plasmids were linearized at ApaL1 and BamH1 sites and on-column purified from agarose gels (Gel Extract II kit, Macherey Nagel). Fertilized eggs from CBA/Ca×C57BL/10 mating were microinjected with the construct, using standard protocols. Transgenic founders carrying the pCX-eGFP-U6-shSLY construct (shSLY mice) were identified by the ubiquitous expression of eGFP. Two female founders with “strong” eGFP expression were obtained (one transgenic for the sh136 construct, the other for the sh367 construct) and crossed with XY^RIII^ males on a random-bred MF1 albino (National Institute for Medical Research colony) background. These females transmitted the transgene and gave rise to two lines of transgenic mice. The lines are maintained by further backcrossing shSLY transgenic females to MF1 mice and generate XY^RIII^ males with (tsgic) and without (neg sib) the transgene. To produce 2/3MSYq^−^ sh367 transgenic mice, sh367 transgenic females were crossed with XY^RIII^qdel males on an MF1 background [12]. The breeding strategy to obtain 9/10MSYq^−^, MSYq^−^, and control mice was described previously [15]. Animal procedures were in accordance with the United Kingdom Animal Scientific Procedures Act 1986 and were subject to local ethical review.

### Elutriation of Spermatids

To obtain cell fractions enriched in spermatids, an adapted protocol from the trypsin method described by Meistrich [53] was used. Testes from a group of four to five adult mice from the same genotype (i.e., three groups of sh367 transgenic mice, three groups of sh367-negative siblings, and two groups of 2/3MSYq^−^) were used for the study. Testes were dissected and chopped in 20 ml of DMEM (GIBCO) and treated with 2.5 mg/ml trypsin (GIBCO) and 50 µg/ml DNase I (Sigma) for 30 min at 31°C with stirring. After adding fetal calf serum (GIBCO) (final concentration of 8%), the cells were passed through a 100-µm filter. Cells were then centrifuged at room temperature (500 *g*, 15 min), resuspended in DMEM 0.5% bovine serum albumin (Sigma) with 50 µg/ml DNase I, and cooled on ice. Cells were counted and checked for clumps before proceeding with the elutriation. Cell integrity was checked using Trypan blue. Fractions enriched in different testis cell types were separated with a JE-6B elutriator (Beckman) with conditions described before [54]. Collected fractions were washed in PBS, and cell pellets were frozen down at −80°C. Fraction content was assessed based on cell morphology after DAPI staining. Fractions #6 contained >90% round spermatids and were used for RNA and protein analyses.

### Northern Blotting

Transgenically delivered shRNAs were detected using the Northern blot protocol optimized for short transcripts described by Shukla et al. [55]. Sh136 reverse primer and sh367 reverse primers were used as probes to detect sh136 and sh367 RNAs. All sequences are available in Table S3.

### Western Blot and Immunofluorescence

Western blot analyses were performed as described previously [18]. Briefly, 10 to 15 µg of testis or spermatid fraction protein extracts were run on a 12% SDS/polyacrylamide gel. Following transfer and blocking, membranes were incubated overnight with a primary antibody (anti-SLX antibody [18] and anti-SLY antibody [17] were used at 1/3,000; anti-SSTY1 antibody, i.e., anti-YMT2b [32], and anti-DKKL1 [R&D Systems] were used at 1/1,000 and anti-β-actin [Sigma] at 1/50,000). Incubation with the corresponding secondary antibody, coupled to peroxidase and detection by chemiluminescence, were carried out as described by the manufacturer (SuperSignal West Pico, Pierce).

Immunofluorescence experiments were performed on testis material fixed in 4% buffered paraformaldehyde as described previously [18]. Anti-SLX [18], anti-DKKL1 (R&D Systems), and anti-SLY [17] antibodies were used at 1/100, and a preimmune rabbit serum was used as a control. For nuclear detection of SLY, an additional step of 15-min permeabilization with 0.5% Triton X-100 (Sigma) was performed prior to antigen retrieval, and blocking was performed using 5% fetal calf serum (GIBCO). Alexa Fluor 594–conjugated peanut agglutinin lectin (Invitrogen) was used to stage the testis tubules [56].

### Antibody Detection and Chromosome Painting on Surface-Spread Testicular Cells

A portion of testis (approximately 25 mg) was chopped in 1 ml of RPMI medium (GIBCO) and transferred to a round-bottomed tube. Five milliliters of fixative solution (2.6 mM sucrose, 1.86% formaldehyde) were added to the cells drop by drop, and cells were mixed by inverting the test tube three times. The cell suspension was incubated at room temperature for 5 min before proceeding to centrifugation (1,200 rpm, 8 min). The fixative was then removed and the cells resuspended in six drops of PBS (GIBCO). Two drops of the cell suspension were spread on Superfrost Plus slides (BHD) and air dried for 2 min. The cells were permeabilized by adding 0.5% Triton X-100 to the slides for 10 min, and washed twice in PBS before incubation in blocking buffer (PBS, 0.15% BSA, 0.1% Tween-20) for 30 min at room temperature in a humid chamber. Incubation with the primary antibody (anti-SLY [17], anti-CBX1, or anti-H3K9me3 [Upstate] diluted 1/100) was carried out for 2 h in a humid chamber at 37°C. Three washes of 2 min in PBS were performed before proceeding with secondary antibody detection as described previously [17]. As a control for specificity, SLY antibody was preabsorbed with 8 µg of SLY immunogenic peptide or with 8 µg of a noncompeting peptide (SLX peptide [18]). Controls are described in Figure S10.

For quantification of CBX1 or H3K9me3 signals, the signal intensity over the PMSC and over the chromocenter was measured using the DeltaVision SoftWoRx software, and PMSC/chromocenter ratio was calculated for each cell. (See Figure S8.)

Chromosome painting was performed as described previously [57].

### Histology and Analysis of Sperm Head Morphology

Testes were fixed in Bouin (Sigma) and wax-embedded. Five-micron sections were stained with periodic acid–Schiff (PAS). For the analysis of the sperm shedding delay, ten tubules of stage IX to XI were analyzed per mouse, for five mice per genotype. Silver staining of sperm smears obtained from the initial caput epididymis was performed as described previously [11].

### In Vitro Fertilization and Fertility Testing

In vitro fertilization (IVF) was performed with sperm from three sh367 transgenic males and three negative controls, using oocytes from B6D2F1 (C57BL/6×DBA/2) hybrid and MF1 outbred females. Each male was tested in duplicate following initial semicastration. In each IVF session, sperm from each male were incubated in parallel with oocytes from the two types of females. The method for IVF has been reported before [28]. Briefly, sperm were released from a single epididymis directly into T6 medium and capacitated for 1.5 h, prior to addition of the oocytes-cumulus complexes obtained from hormonally stimulated females. The gametes were co-incubated for 4 h with sperm density ∼2–3×10^6^/ml. After fertilization, the oocytes were washed and cultured; the number of two-cell embryos was recorded after 24 h.

To analyze sperm number and motility, a small portion of sperm suspension was placed in a hemacytometer chamber. Three independent scorings were done per sample, and the final result was a mean of these scorings.

The fertility of shSLY mice was assessed over a period of 7 mo by mating two sh367 transgenic males and two negative siblings with MF1 females. Mating was confirmed by the presence of copulatory plugs.

### Real-Time PCR and Microarray Analyses

For real-time reverse transcription-polymerase chain reaction (RT-PCR), total testis RNA was extracted using Trizol and then DNaseI-treated (Invitrogen). Reverse transcription of polyadenylated RNA was performed with Superscript Reverse Transcriptase II, according to the manufacturer's guidelines (Invitrogen). Real-time PCR was performed using Absolute qPCR SYBR Green ROX mix (ThermoFisher) on an ABI PRISM 7500 machine (Applied Biosystems). PCR reactions were incubated at 95°C for 15 min followed by 40 PCR cycles (5 s at 95°C, 20 s at 60°C, and 45 s at 68°C). Primer sequences are available in Table S3. Samples from four transgenic mice and three nontransgenic siblings (negative controls), all at 2 mo of age, were analyzed. All reactions were carried out in triplicate per assay, and β-actin was included on every plate as a loading control. The difference in PCR cycles with respect to β-actin (ΔCt) for a given experimental sample was subtracted from the mean ΔCt of the reference samples (negative siblings) (ΔΔCt). For the quantification of *Sly* knock-down, values were further normalized to ΔΔCt values of the spermatid-specific control *Acrv1*. This was to have a more robust analysis when compared with 2/3MSYq^−^ mice, which have variability in spermatid content.

For microarray analyses, absolute expression values were obtained by single-color hybridizations (Illumina BeadChip, mouse whole-genome array, v2) for three sh367 transgenic individuals and matched littermate controls (negative siblings), and RNA from each individual was hybridized separately. A similar analysis was performed on 2/3MSYq^−^, 9/10MSYq^−^, and MSYq^−^ samples and appropriate age/strain-matched controls. In each case, pooled RNA from two or three individuals was used as the sample. Differentially expressed genes were grouped into five categories based on their expression ratios across all genotypes (see Figure S5). Similar microarray analyses were performed on juvenile testes (17 d postpartum) from three sh367 males and three littermate controls (negative siblings). There was no significant change of gene expression between the two groups. Microarray analyses were also performed on purified spermatid fractions from two groups of sh367 transgenic mice, two groups of sh367 negative siblings, and two groups of 2/3MSYq^−^.

### Statistical Analysis

For comparisons of the incidence of sperm head abnormalities and of sperm motility (after conversion of percentages to angles), and of the CBX1 and H3K9me3 PMSC/chromocenter intensity ratios, differences between genotypes were assessed by ANOVA using the Generalized Linear Model provided by NCSS statistical data analysis software. Chi-square analysis was used to compare sex ratio and IVF data; for the sex ratio, we used a one-tailed test of significance since we sought to test whether there was a sex ratio distortion in favor of females (as seen in 2/3MSYq^−^ mice). Student *t*-test was used to compare the data obtained for fecundity, sperm number, testis weight, Northern and Western blot quantification, and real-time PCR (performed on the ΔΔCt values). For microarray analysis, quantile normalization of all expression data was performed using BeadStudio (Illumina). Data for the normal/mutant sh367 animals were compared in BeadStudio, using the Illumina custom error model with a false discovery rate of 5%. For the cluster analysis, normalized data for all samples were imported into Inforsense Discovery Studio (Inforsense), log_2_-transformed, and expression ratios calculated relative to the appropriate controls. Hierarchical clustering was then performed on the ratio values, using Pearson correlation as the distance metric.

## Supporting Information

Figure S1 **Tests of the efficiency and specificity of shSLY constructs in HEK293 cells.** (A) Western blot detection of SLY and GFP proteins in HEK293 cells cotransfected with SLY-expressing vector (pCMV-SLY) and one pCX-eGFP-shRNA construct (sh136, sh367, or shIRR). shIRR construct has the same structure as sh136 and sh367 but expresses an irrelevant shRNA sequence (sequence available in Table S3). Levels of expression were quantified using ImageJ software and normalized with respect to eGFP (loading and transfection control). The values plotted on the graphs are the percentage of expression of SLY protein (±standard errors) in sh136- or sh367-transfected cells compared to cells transfected with shIRR. These results show that sh136 and sh367 constructs efficiently knock down the expression of SLY in cells. (B) Same as above, except that HEK293 cells were cotransfected with SLX-expressing vector (pCMV-SLX) and one pCX-eGFP-shRNA construct. The values plotted on the graphs are the percentage of expression of SLX protein (±standard errors) in sh136- or sh367-transfected cells compared to cells transfected with shIRR. These results show that sh136 and sh367 constructs have no effect on the expression of SLX in cells.
(0.08 MB PDF)

Figure S2 **Western blot detection in purified round spermatids.** (A) Detection of SLY, SSTY1, SLX, and DKKL1 in purified round spermatids of *Sly*-deficient mice (sh367 tsgic) and controls (neg sib). Actin detection was used as a loading control. No SLY protein could be observed in *Sly*-deficient round spermatids (sh367 tsgic). (B) Levels of expression were quantified using ImageJ software and normalized with respect to actin signal. SLX and SSTY1 proteins, encoded respectively by X and Y genes found to be up-regulated at the transcript level, are significantly increased in round spermatids of sh367 transgenic mice compared to control, whereas the DKKL1 level is unchanged. Statistical significance with respect to corresponding control: * *p*≤0.05 (*t*-test).
(0.11 MB PDF)

Figure S3 **Tests for potential “off target” effects of the RNA interference.** (A) Detection of mir-t3 and mir-t25 by Northern blots in testis from shSLY transgenic mice (sh367 tsgic) and controls (neg sib). U6snRNA detection was used as loading control. The graphs show the quantification of mir-t3 and mir-t25 level of expression from Northern blot films, using ImageJ software. The plotted values represent the level of expression normalized to U6snRNA level and to the average value obtained for the negative siblings (±standard errors). No difference of mir-t3 or mir-t25 expression could be observed in testes of sh367 transgenic mice and controls. (B) Quantification by real-time PCR of transcript levels of the interferon pathway target gene *Oas1b* (2′,5′-oligoadenylate synthetase). The *y*-axis indicates the level of expression normalized to actin level and to the average value obtained for the negative siblings (2^ΔΔCt^ ± standard errors). No change in *Oas1b* expression level could be detected in testes of shSLY mice (both sh136 and sh367) compared to control (neg sib).
(0.14 MB PDF)

Figure S4 **Detailed analysis of sperm head abnormalities in 2/3MSYq^−^ sh367 transgenic mice.** Bar graph representing the percentage of slightly flattened, grossly flattened, and other gross sperm head abnormalities in 2/3MSYq^−^ sh367 transgenic mice compared to sh367 transgenic mice with a normal Y^RIII^ chromosome (sh367 tsgic) and compared to 2/3MSYq^−^ nontransgenic mice. The presence of the sh367 transgene in the context of 2/3MSYq^−^ significantly increases the percentage of grossly flattened and other gross sperm head abnormalities in comparison to 2/3MSYq^−^ nontransgenic mice. There is also a significant increase of other gross sperm head abnormalities between 2/3MSYq^−^ sh367 transgenic mice and sh367 transgenic mice with a normal Y^RIII^ chromosome. One or two asterisks indicate significant difference between two samples (respectively, *p*<0.05 or *p*<0.001; ANOVA).
(0.07 MB PDF)

Figure S5 **Results** **from the microarray analyses performed on adult testes of** ***Sly*** **-deficient mice (sh367) and MSYq-deficient mice (2/3MSYq^−^, 9/10MSYq^−^, and MSYq^−^).** Differentially expressed genes were grouped into five categories based on their expression ratios across all genotypes. The front page of the Excel sheet shows the heat map for this cluster analysis, together with the chromosomal distribution of the genes in each category. The second page shows the normalized expression values and annotation data for the 230 Illumina probes detected as differentially expressed in sh367 testis RNA relative to RNA from normal littermates.
(0.14 MB XLS)

Figure S6 **Results** **from the microarray analyses performed on purified spermatids from** ***Sly*** **-deficient mice (sh367) and MSYq-deficient mice (2/3MSYq^−^).** Differentially expressed genes were grouped into seven categories based on their expression ratios across genotypes. The front page of the Excel sheet shows the chromosomal distribution of the genes in each category. The second page shows the normalized expression values and annotation data for the 272 Illumina probes detected as differentially expressed (with a change >1.5-fold) in sh367 spermatid RNA relative to RNA from normal littermates. The table on the third page shows the number of genes identified in the whole testis array that are also significant in the spermatid array.
(0.10 MB XLS)

Figure S7 **Detection of SLX protein by immunofluorescence in testis sections of shSLY mice and negative siblings.** SLX antibody and the rabbit preimmune serum were detected in green; DAPI (in blue) was used to stain nuclei. Pictures were taken using the same image capture parameters. Similar results were obtained for sh136 mice. (A) Testis tubules under low magnification (scale bars indicate 45 µm). (B) and (C) Testis tubules under high magnification (scale bars indicate 10 µm). The level of SLX protein is higher in *Sly*-deficient mice compared to control, but its site of expression is the same (spermatid-specific).
(0.19 MB PDF)

Figure S8 **Measurement of the intensity of H3K9me3 and CBX1 staining over PMSC in surface-spread spermatids.** (A) Graph representing the distribution of the H3K9me3 PMSC signal intensity per spermatid. The average values obtained for *Sly*-deficient mice (i.e., sh367 tsgic) and wild-type mice (i.e., sh367 neg sib) are, respectively, 0.44 and 0.58 (*p*<0.005; ANOVA test). (B) Graph representing the distribution of the CBX1 PMSC signal intensity per spermatid. The average values obtained for *Sly*-deficient mice (i.e., sh367 tsgic) and wild-type mice (i.e., sh367 neg sib) are, respectively, 0.29 and 0.36 (*p*<0.05; ANOVA test). (C) Table indicating the percentage of spermatids with reduced (below average) and normal (above average) PMSC signal of H3K9me3 and CBX1. *Sly*-deficient spermatids have a higher proportion of spermatids with reduced H3K9me3 and CBX1 staining over PMSC.
(0.11 MB PDF)

Figure S9 **Detection of SLY protein by immunofluorescence in testis tubules.** (A) and (B) Stage I to V testis tubules and stage VII to XI testis tubules, under low magnification (scale bars indicate 20 µm); (C) stage VII, IX, and X tubules under high magnification (scale bars indicate 10 µm). Pictures were taken using the same image capture parameters. Sections from wild-type (wt) testis tubules were compared to sections from *Sly*-deficient mice (shSLY). DAPI (in white or in blue in the merged picture) was used to stain nuclei. Lectin-PNA (in red) was used to determine tubule stage. SLY protein (in green) is detected in the spermatid nuclei from stage II–III until stage VIII. At the onset of elongation (stage IX), SLY protein is excluded from the spermatid nuclei. At higher magnification, SLY protein seems to accumulate on postmeiotic sex chromatin. Immunodetection of SLY in *Sly*-deficient testis tubules did not give any specific signal, and was used as a negative control.
(0.60 MB PDF)

Figure S10 **Detection of SLY protein by immunofluorescence in surface-spread spermatids.** DAPI (in blue) was used to stain nuclei. SLY protein (in green) colocalizes with PMSC in wild-type (wt) round spermatids (cf. also Figure 8). When the antibody was preabsorbed with SLY peptide, the signal disappeared. When the antibody was preabsorbed with a noncompeting peptide, SLY antibody signal was maintained and colocalized with PMSC. No signal was observed in round spermatids deficient for SLY protein (i.e., sh367 tsgic sample). *Sly*-deficient surface-spread cells can be recognized by the presence of an abnormal sperm head. All these controls demonstrate the specificity of the signal obtained with SLY antibody.
(0.08 MB PDF)

Figure S11 **Detection of DKKL1 protein by immunofluorescence in testis tubules.** (A) and (B) Stage I to III testis tubules and stage V to VIII testis tubules. Scale bars indicate 20 µm. Pictures were taken using the same image capture parameters. Sections from *Sly*-deficient mice (sh367 tsgic) testis tubules were compared to sections from wild-type mice (sh367 neg sib). DKKL1 antibody was detected (in green). Lectin-PNA (in red) was used to determine tubule stage. DKKL1 intracellular localization was similar in *Sly*-deficient mice and wild-type mice, being barely detectable at stage I and clearly localized to the acrosome from stage II–III.
(0.28 MB PDF)

Table S1 **Testis weight, sperm number, and sperm motility of sh367 transgenic mice and controls.** The mean testis weight value has been calculated from the average testis weight per mouse, for at least seven mice per genotype. Sperm numbers have been scored per cauda epididymis.
(0.01 MB PDF)

Table S2 **Breeding data from all sh136 or sh367 transgenic males and negative siblings obtained so far.**
(0.01 MB PDF)

Table S3 **List of the primers used in the study.** Unless otherwise stated, primers were described previously [6],[16],[58]–[61].
(0.09 MB PDF)

## Abbreviations

IVF: in vitro fertilization
MSCI: meiotic sex chromosome inactivation
MSYq: male-specific region of the mouse Y chromosome long arm
PMSC: postmeiotic sex chromatin
PSCR: postmeiotic sex chromatin repression
shRNA: short hairpin RNA
siRNA: small interfering RNA
shSLY: *Sly* short hairpin sequence
