# Supplementary figures and images for: The Multicopy Gene Sly Represses the Sex Chromosomes in the Male Mouse Germline after Meiosis
Source: PLoS Biol. 2009 Nov 17;7(11):e1000244. doi: 10.1371/journal.pbio.1000244 (PMC2770110; doi:10.1371/journal.pbio.1000244)

A

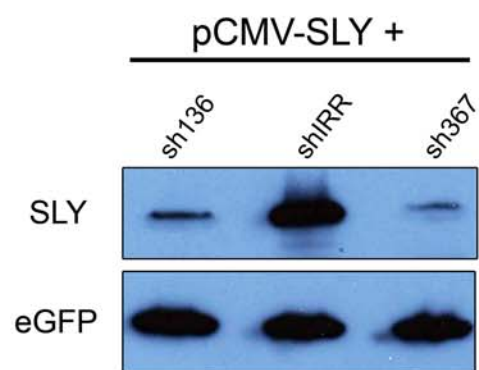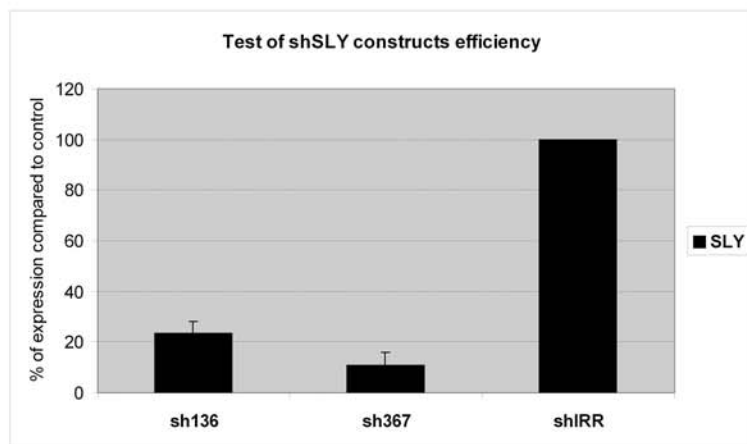

B

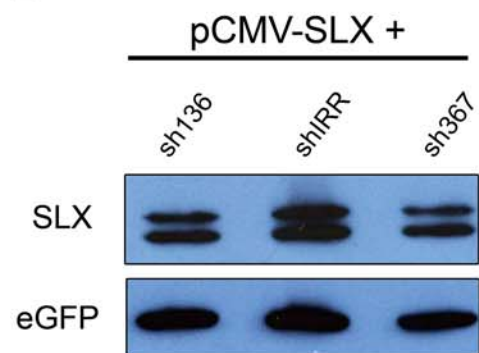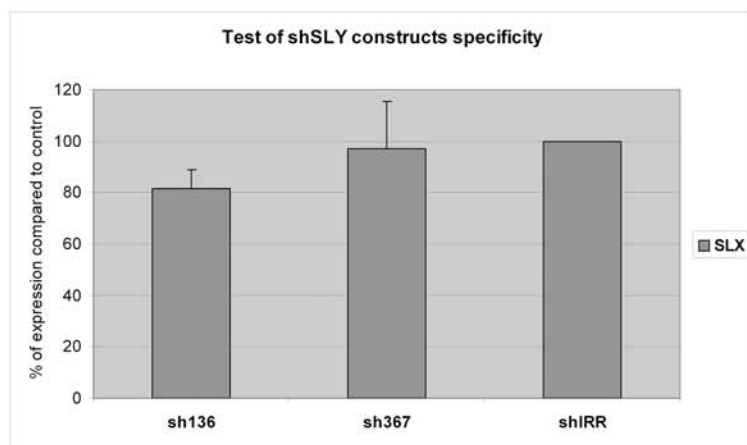

Supplement: Figure S1 — Tests of the efficiency and specificity of shSLY constructs in HEK293 cells. (A) Western blot detection of SLY and GFP proteins in HEK293 cells cotransfected with SLY-expressing vector (pCMV-SLY) and one pCX-eGFP-shRNA construct (sh136, sh367, or shIRR). shIRR construct has the same structure as sh136 and sh367 but expresses an irrelevant shRNA sequence (sequence available in Table S3). Levels of expression were quantified using ImageJ software and normalized with respect to eGFP (loading and transfection control). The values plotted on the graphs are the percentage of expression of SLY protein (±standard errors) in sh136- or sh367-transfected cells compared to cells transfected with shIRR. These results show that sh136 and sh367 constructs efficiently knock down the expression of SLY in cells. (B) Same as above, except that HEK293 cells were cotransfected with SLX-expressing vector (pCMV-SLX) and one pCX-eGFP-shRNA construct. The values plotted on the graphs are the percentage of expression of SLX protein (±standard errors) in sh136- or sh367-transfected cells compared to cells transfected with shIRR. These results show that sh136 and sh367 constructs have no effect on the expression of SLX in cells. (0.08 MB PDF) [file pbio.1000244.s001.pdf]

A

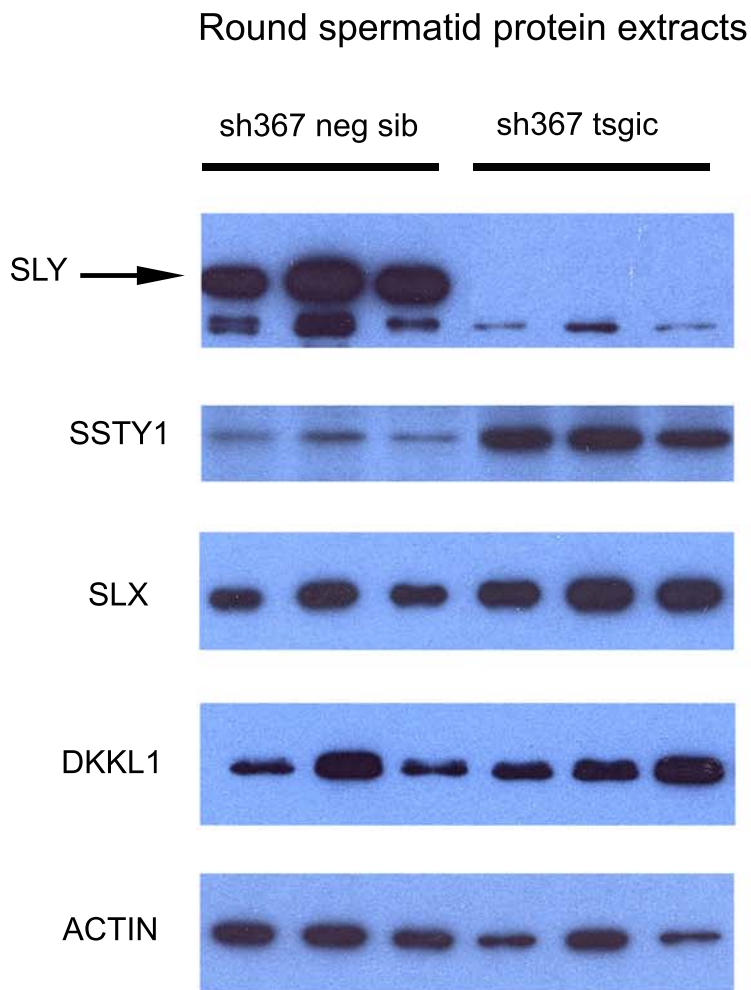

B

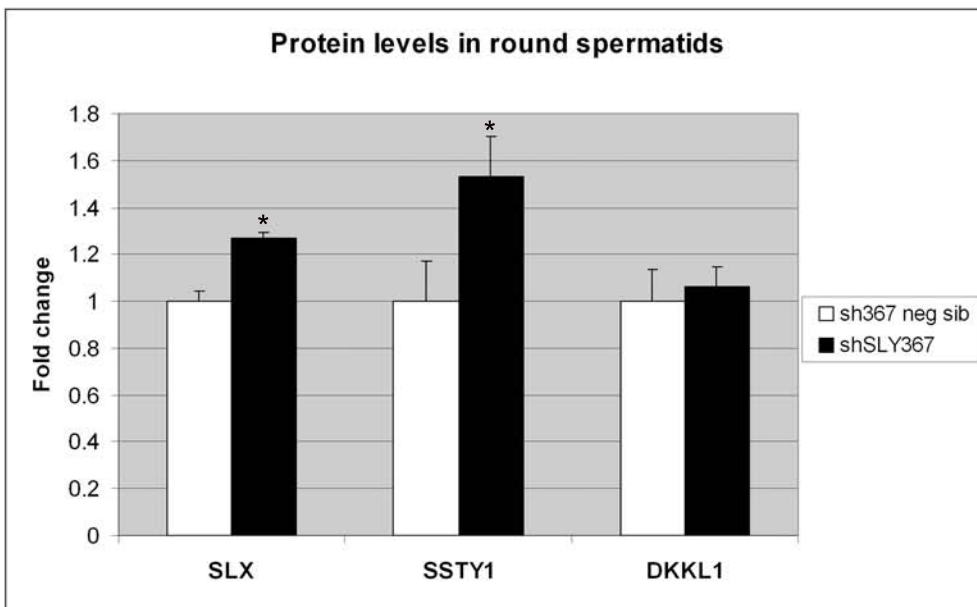

Supplement: Figure S2 — Western blot detection in purified round spermatids. (A) Detection of SLY, SSTY1, SLX, and DKKL1 in purified round spermatids of Sly-deficient mice (sh367 tsgic) and controls (neg sib). Actin detection was used as a loading control. No SLY protein could be observed in Sly-deficient round spermatids (sh367 tsgic). (B) Levels of expression were quantified using ImageJ software and normalized with respect to actin signal. SLX and SSTY1 proteins, encoded respectively by X and Y genes found to be up-regulated at the transcript level, are significantly increased in round spermatids of sh367 transgenic mice compared to control, whereas the DKKL1 level is unchanged. Statistical significance with respect to corresponding control: * p≤0.05 (t-test). (0.11 MB PDF) [file pbio.1000244.s002.pdf]

A

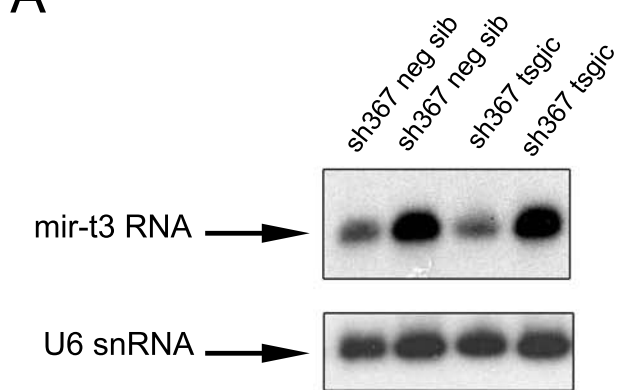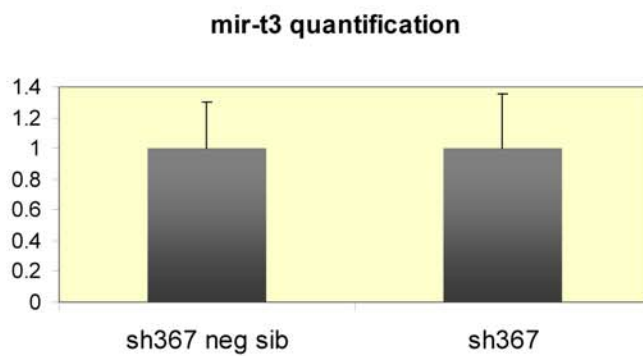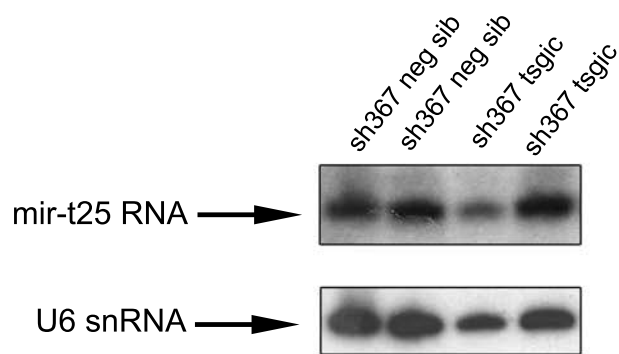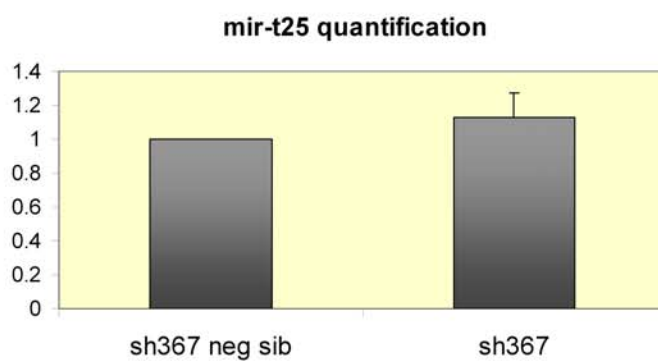

B

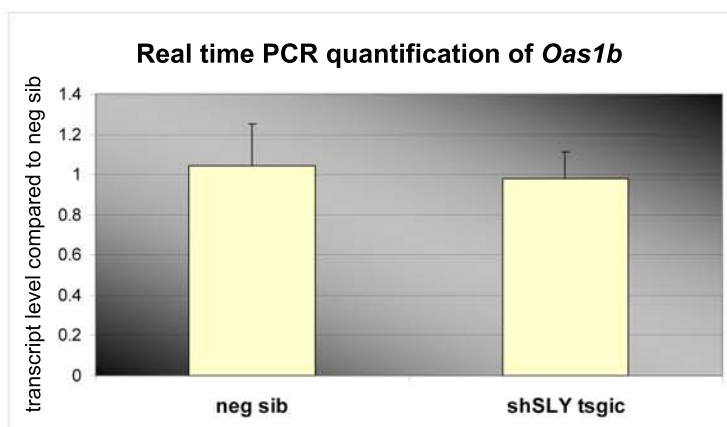

Supplement: Figure S3 — Tests for potential “off target” effects of the RNA interference. (A) Detection of mir-t3 and mir-t25 by Northern blots in testis from shSLY transgenic mice (sh367 tsgic) and controls (neg sib). U6snRNA detection was used as loading control. The graphs show the quantification of mir-t3 and mir-t25 level of expression from Northern blot films, using ImageJ software. The plotted values represent the level of expression normalized to U6snRNA level and to the average value obtained for the negative siblings (±standard errors). No difference of mir-t3 or mir-t25 expression could be observed in testes of sh367 transgenic mice and controls. (B) Quantification by real-time PCR of transcript levels of the interferon pathway target gene Oas1b (2′,5′-oligoadenylate synthetase). The y-axis indicates the level of expression normalized to actin level and to the average value obtained for the negative siblings (2ΔΔCt ± standard errors). No change in Oas1b expression level could be detected in testes of shSLY mice (both sh136 and sh367) compared to control (neg sib). (0.14 MB PDF) [file pbio.1000244.s003.pdf]

**% of sperm head abnormalities**

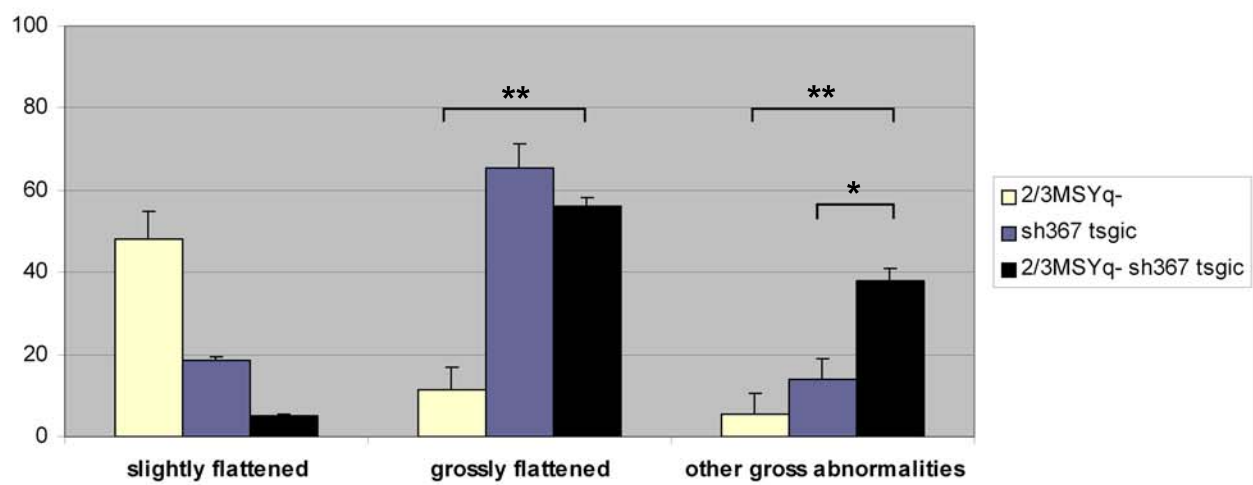

Supplement: Figure S4 — Detailed analysis of sperm head abnormalities in 2/3MSYq− sh367 transgenic mice. Bar graph representing the percentage of slightly flattened, grossly flattened, and other gross sperm head abnormalities in 2/3MSYq− sh367 transgenic mice compared to sh367 transgenic mice with a normal YRIII chromosome (sh367 tsgic) and compared to 2/3MSYq− nontransgenic mice. The presence of the sh367 transgene in the context of 2/3MSYq− significantly increases the percentage of grossly flattened and other gross sperm head abnormalities in comparison to 2/3MSYq− nontransgenic mice. There is also a significant increase of other gross sperm head abnormalities between 2/3MSYq− sh367 transgenic mice and sh367 transgenic mice with a normal YRIII chromosome. One or two asterisks indicate significant difference between two samples (respectively, p<0.05 or p<0.001; ANOVA). (0.07 MB PDF) [file pbio.1000244.s004.pdf]

**A**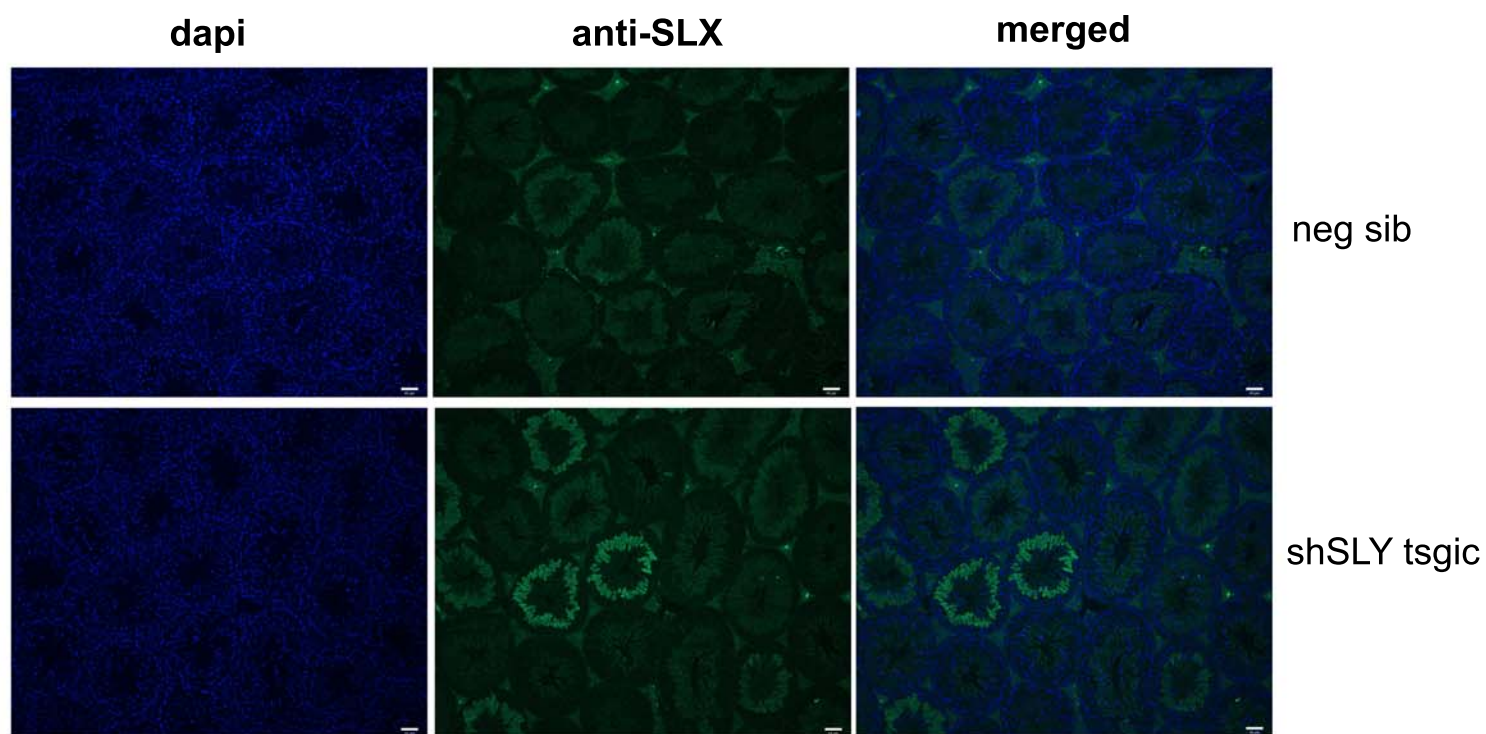**B**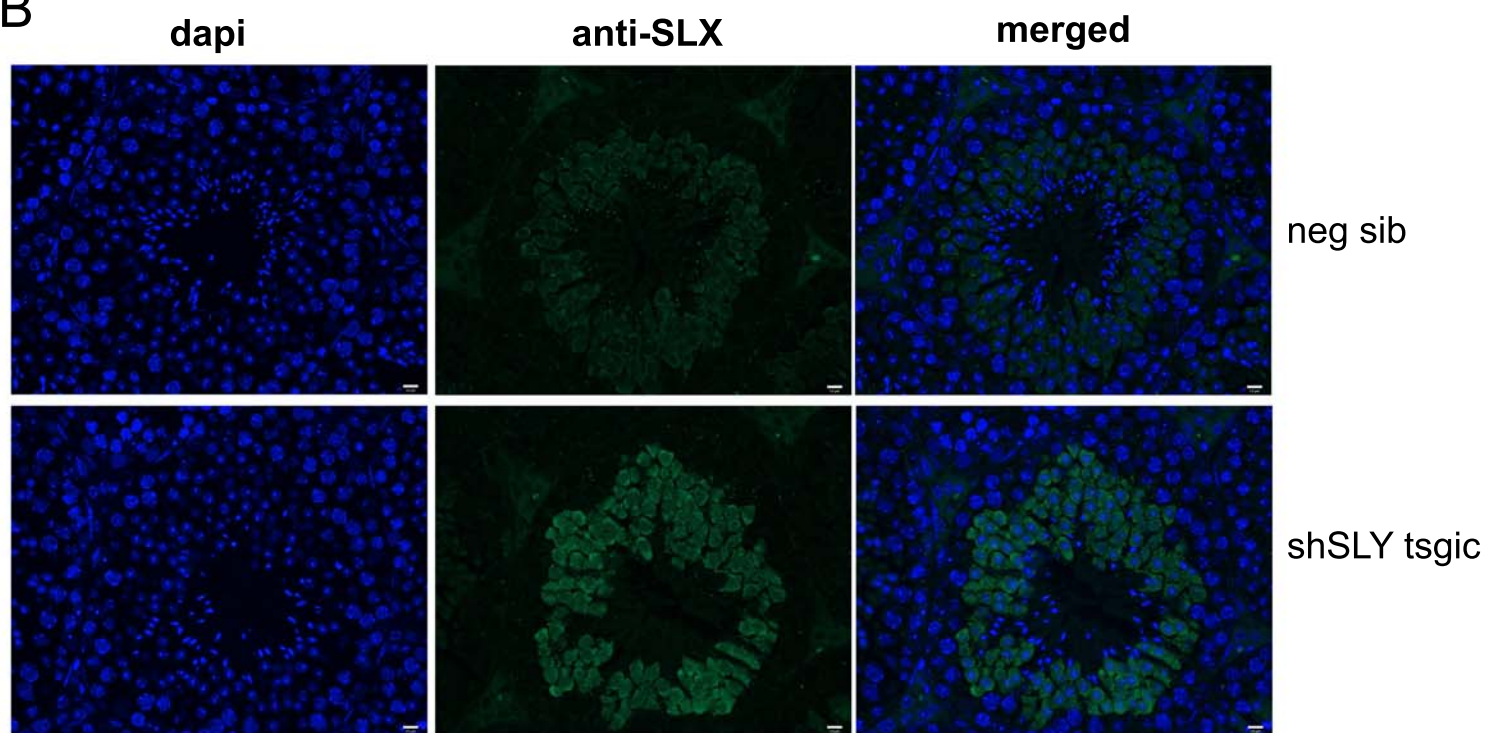**C**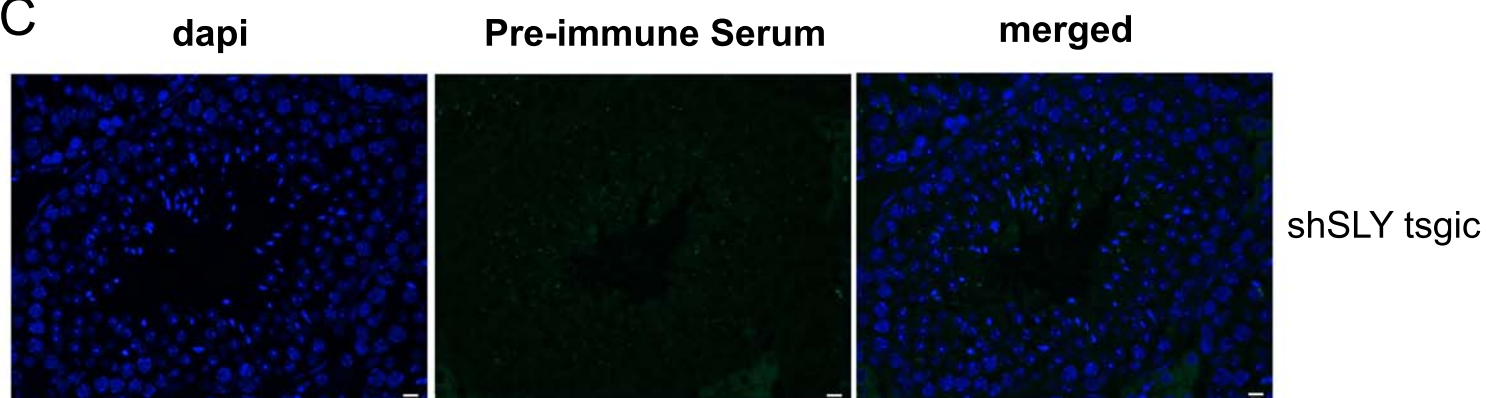

Supplement: Figure S7 — Detection of SLX protein by immunofluorescence in testis sections of shSLY mice and negative siblings. SLX antibody and the rabbit preimmune serum were detected in green; DAPI (in blue) was used to stain nuclei. Pictures were taken using the same image capture parameters. Similar results were obtained for sh136 mice. (A) Testis tubules under low magnification (scale bars indicate 45 µm). (B) and (C) Testis tubules under high magnification (scale bars indicate 10 µm). The level of SLX protein is higher in Sly-deficient mice compared to control, but its site of expression is the same (spermatid-specific). (0.19 MB PDF) [file pbio.1000244.s007.pdf]

DAPI

SLY + DAPI

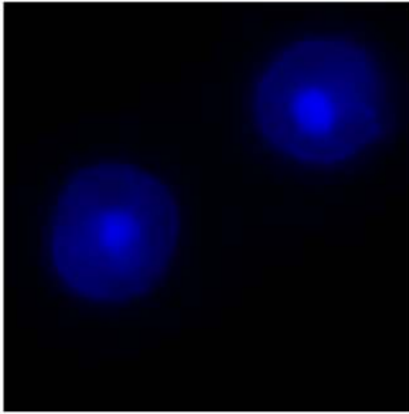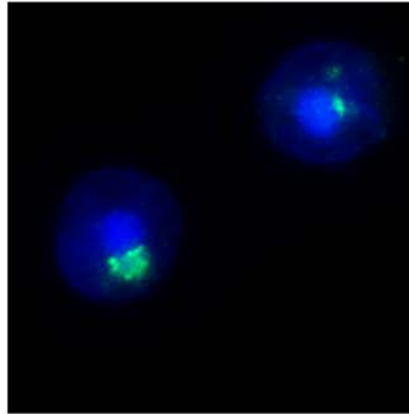

WT

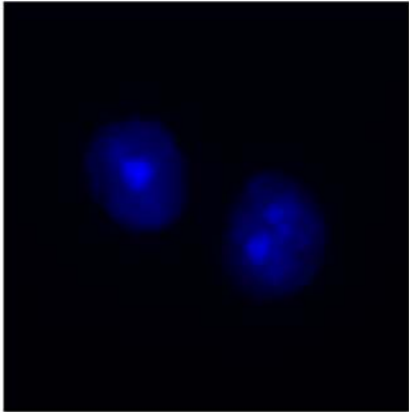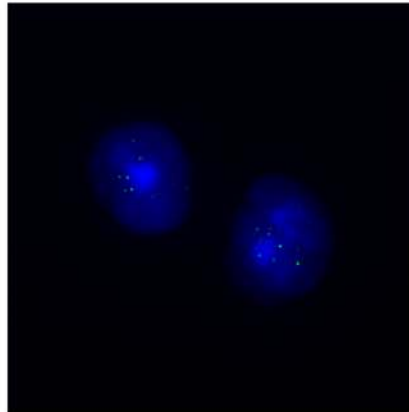

WT+SLY peptide

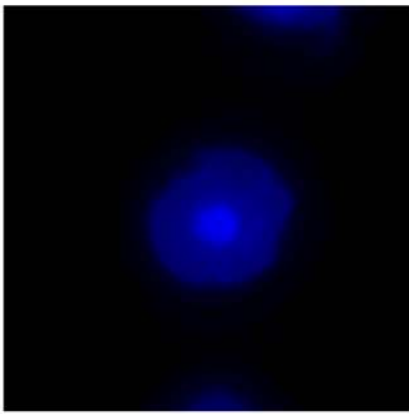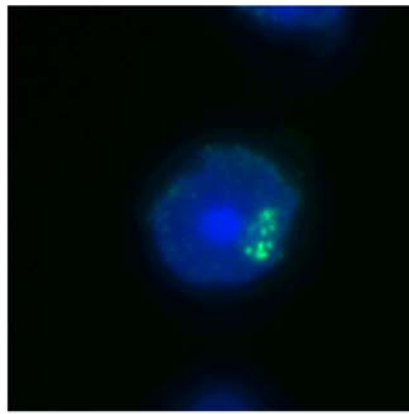

WT+non competing peptide

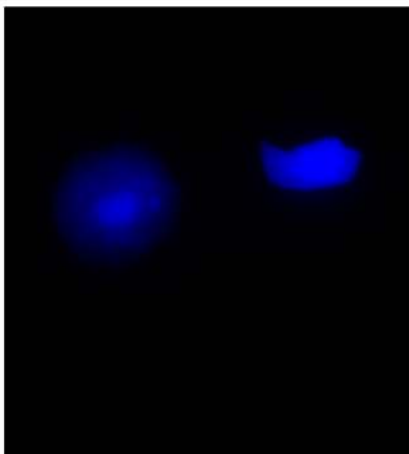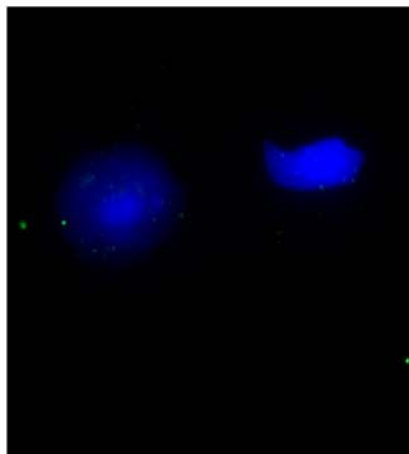

sh367 tsgic

Supplement: Figure S10 — Detection of SLY protein by immunofluorescence in surface-spread spermatids. DAPI (in blue) was used to stain nuclei. SLY protein (in green) colocalizes with PMSC in wild-type (wt) round spermatids (cf. also Figure 8). When the antibody was preabsorbed with SLY peptide, the signal disappeared. When the antibody was preabsorbed with a noncompeting peptide, SLY antibody signal was maintained and colocalized with PMSC. No signal was observed in round spermatids deficient for SLY protein (i.e., sh367 tsgic sample). Sly-deficient surface-spread cells can be recognized by the presence of an abnormal sperm head. All these controls demonstrate the specificity of the signal obtained with SLY antibody. (0.08 MB PDF) [file pbio.1000244.s010.pdf]

A

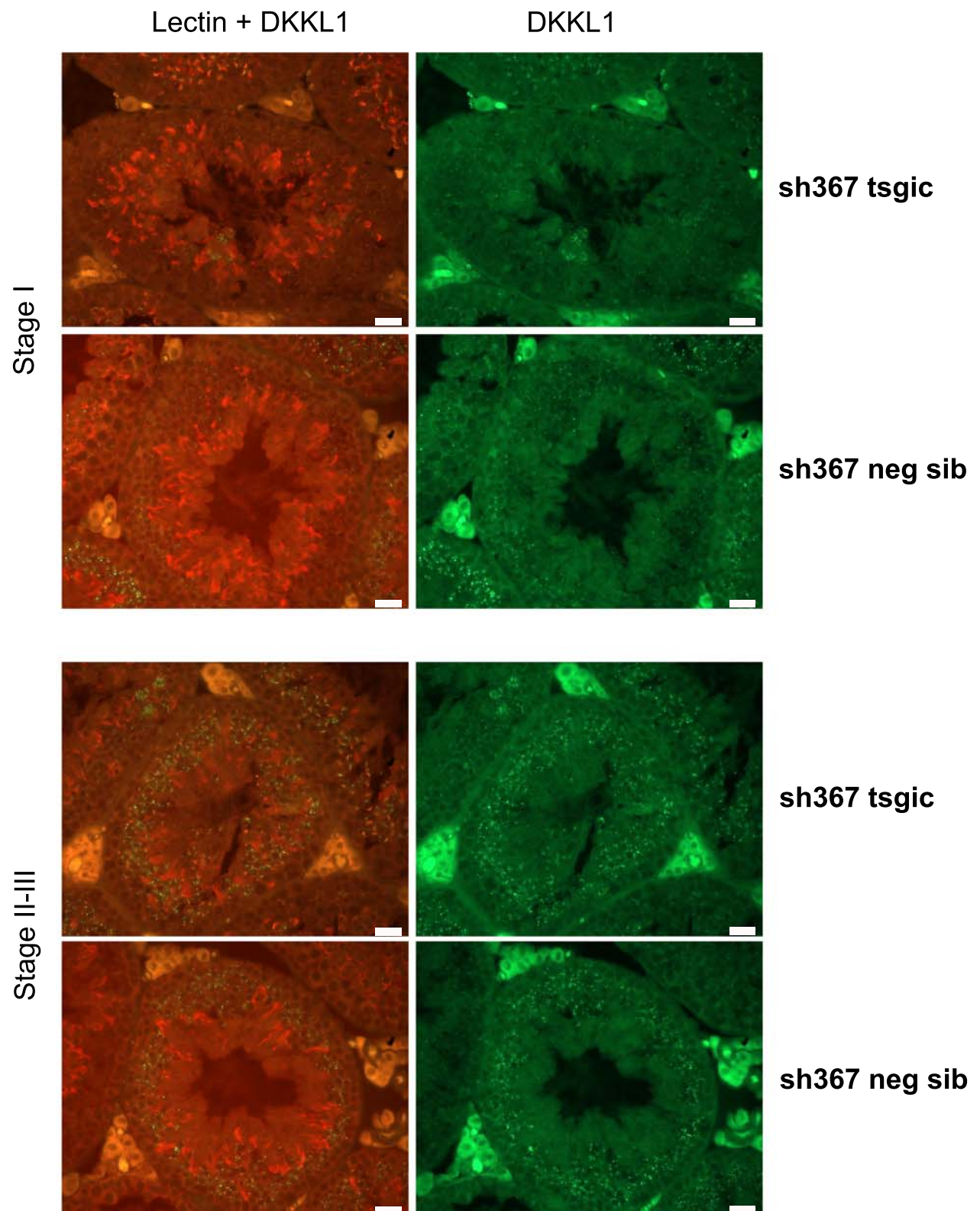

**B**

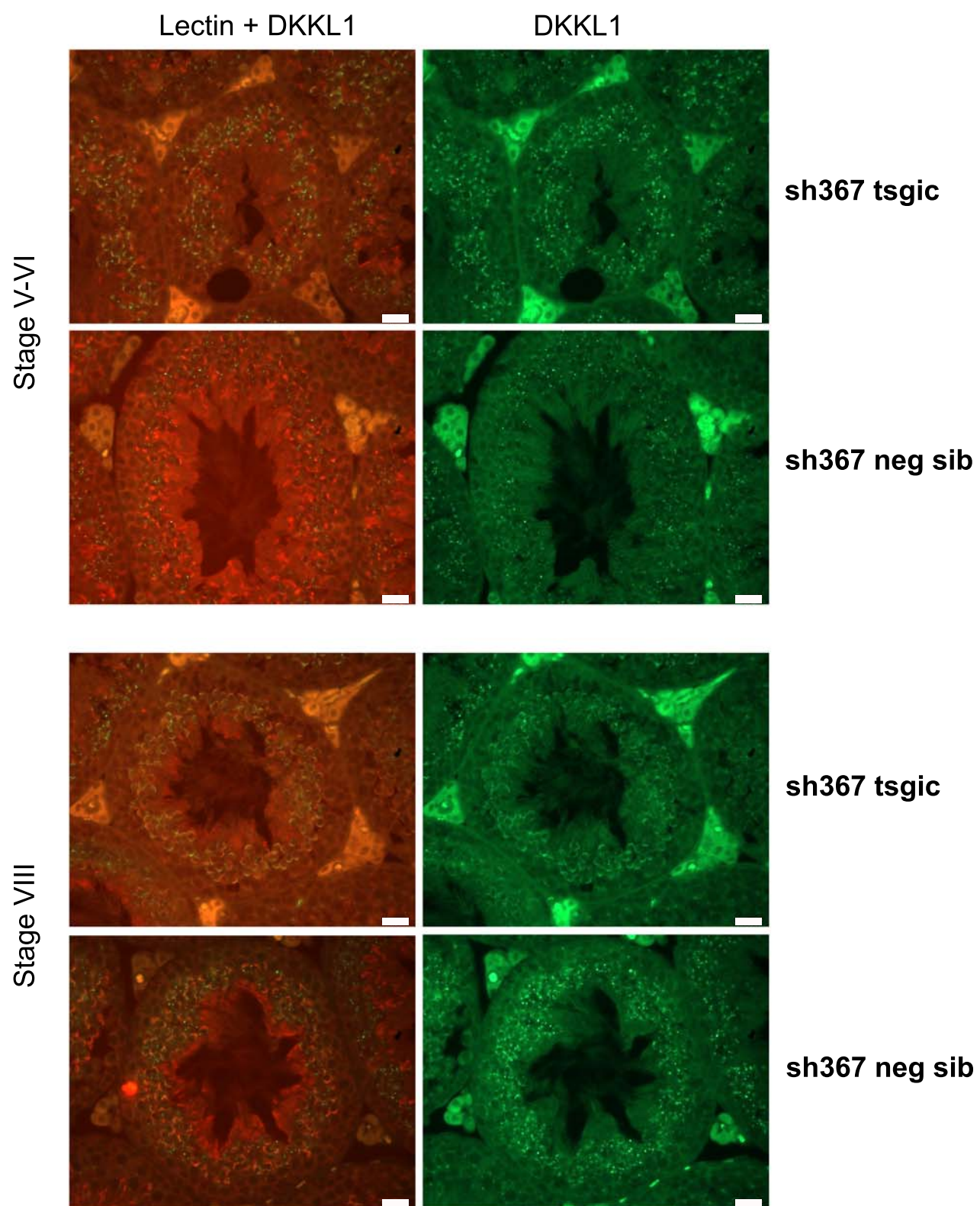

Supplement: Figure S11 — Detection of DKKL1 protein by immunofluorescence in testis tubules. (A) and (B) Stage I to III testis tubules and stage V to VIII testis tubules. Scale bars indicate 20 µm. Pictures were taken using the same image capture parameters. Sections from Sly-deficient mice (sh367 tsgic) testis tubules were compared to sections from wild-type mice (sh367 neg sib). DKKL1 antibody was detected (in green). Lectin-PNA (in red) was used to determine tubule stage. DKKL1 intracellular localization was similar in Sly-deficient mice and wild-type mice, being barely detectable at stage I and clearly localized to the acrosome from stage II–III. (0.28 MB PDF) [file pbio.1000244.s011.pdf]
