# Supplementary material for: The Multicopy Gene Sly Represses the Sex Chromosomes in the Male Mouse Germline after Meiosis
Source: PLoS Biol. 2009 Nov 17;7(11):e1000244. doi: 10.1371/journal.pbio.1000244 (PMC2770110; doi:10.1371/journal.pbio.1000244)
Supplement: Figure S8 — Measurement of the intensity of H3K9me3 and CBX1 staining over PMSC in surface-spread spermatids. (A) Graph representing the distribution of the H3K9me3 PMSC signal intensity per spermatid. The average values obtained for Sly-deficient mice (i.e., sh367 tsgic) and wild-type mice (i.e., sh367 neg sib) are, respectively, 0.44 and 0.58 (p<0.005; ANOVA test). (B) Graph representing the distribution of the CBX1 PMSC signal intensity per spermatid. The average values obtained for Sly-deficient mice (i.e., sh367 tsgic) and wild-type mice (i.e., sh367 neg sib) are, respectively, 0.29 and 0.36 (p<0.05; ANOVA test). (C) Table indicating the percentage of spermatids with reduced (below average) and normal (above average) PMSC signal of H3K9me3 and CBX1. Sly-deficient spermatids have a higher proportion of spermatids with reduced H3K9me3 and CBX1 staining over PMSC. (0.11 MB PDF) [file pbio.1000244.s008.pdf]

A

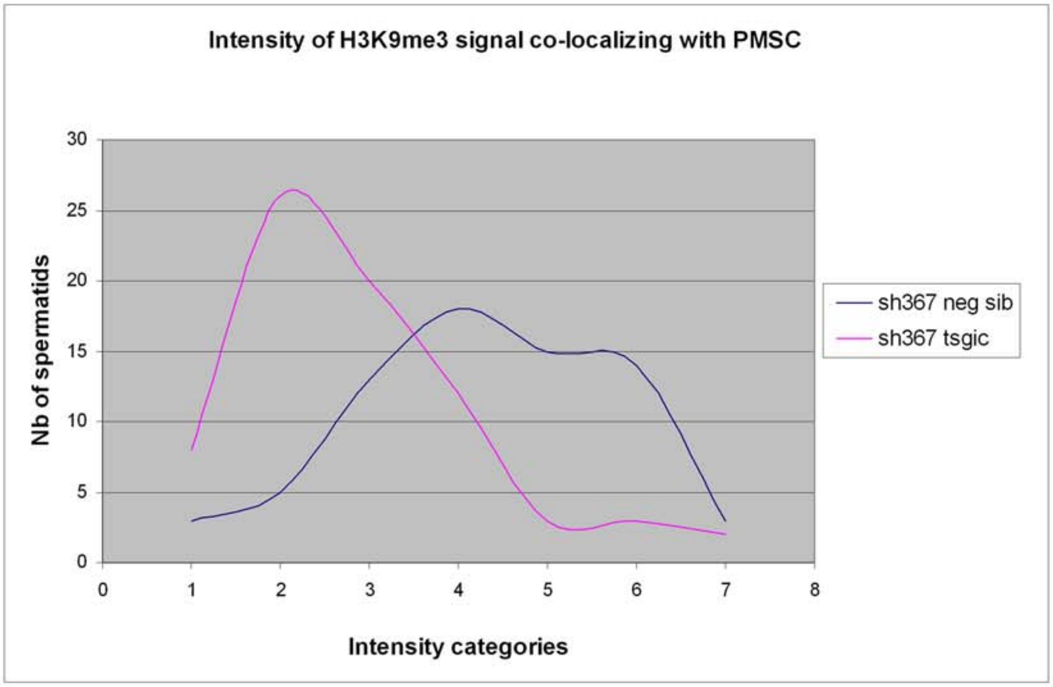

B

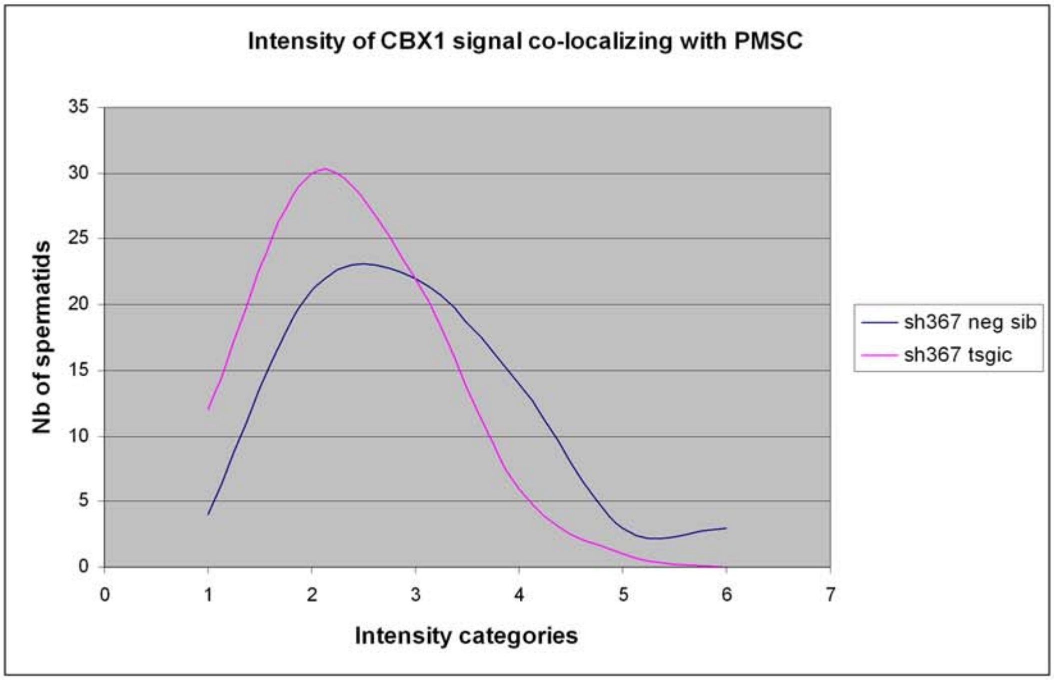

C

	genotype	% of spermatids with reduced PMSC signal	% of spermatids with above average PMSC signal
H3K9me3	sh367 neg sib	46.50%	53.50%
	sh367 tsgic	85%	15%
CBX1	sh367 neg sib	57%	43%
	sh367 tsgic	82%	18%
