# Supplementary material for: The Multicopy Gene Sly Represses the Sex Chromosomes in the Male Mouse Germline after Meiosis
Source: PLoS Biol. 2009 Nov 17;7(11):e1000244. doi: 10.1371/journal.pbio.1000244 (PMC2770110; doi:10.1371/journal.pbio.1000244)
Supplement: Figure S9 — Detection of SLY protein by immunofluorescence in testis tubules. (A) and (B) Stage I to V testis tubules and stage VII to XI testis tubules, under low magnification (scale bars indicate 20 µm); (C) stage VII, IX, and X tubules under high magnification (scale bars indicate 10 µm). Pictures were taken using the same image capture parameters. Sections from wild-type (wt) testis tubules were compared to sections from Sly-deficient mice (shSLY). DAPI (in white or in blue in the merged picture) was used to stain nuclei. Lectin-PNA (in red) was used to determine tubule stage. SLY protein (in green) is detected in the spermatid nuclei from stage II–III until stage VIII. At the onset of elongation (stage IX), SLY protein is excluded from the spermatid nuclei. At higher magnification, SLY protein seems to accumulate on postmeiotic sex chromatin. Immunodetection of SLY in Sly-deficient testis tubules did not give any specific signal, and was used as a negative control. (0.60 MB PDF) [file pbio.1000244.s009.pdf]

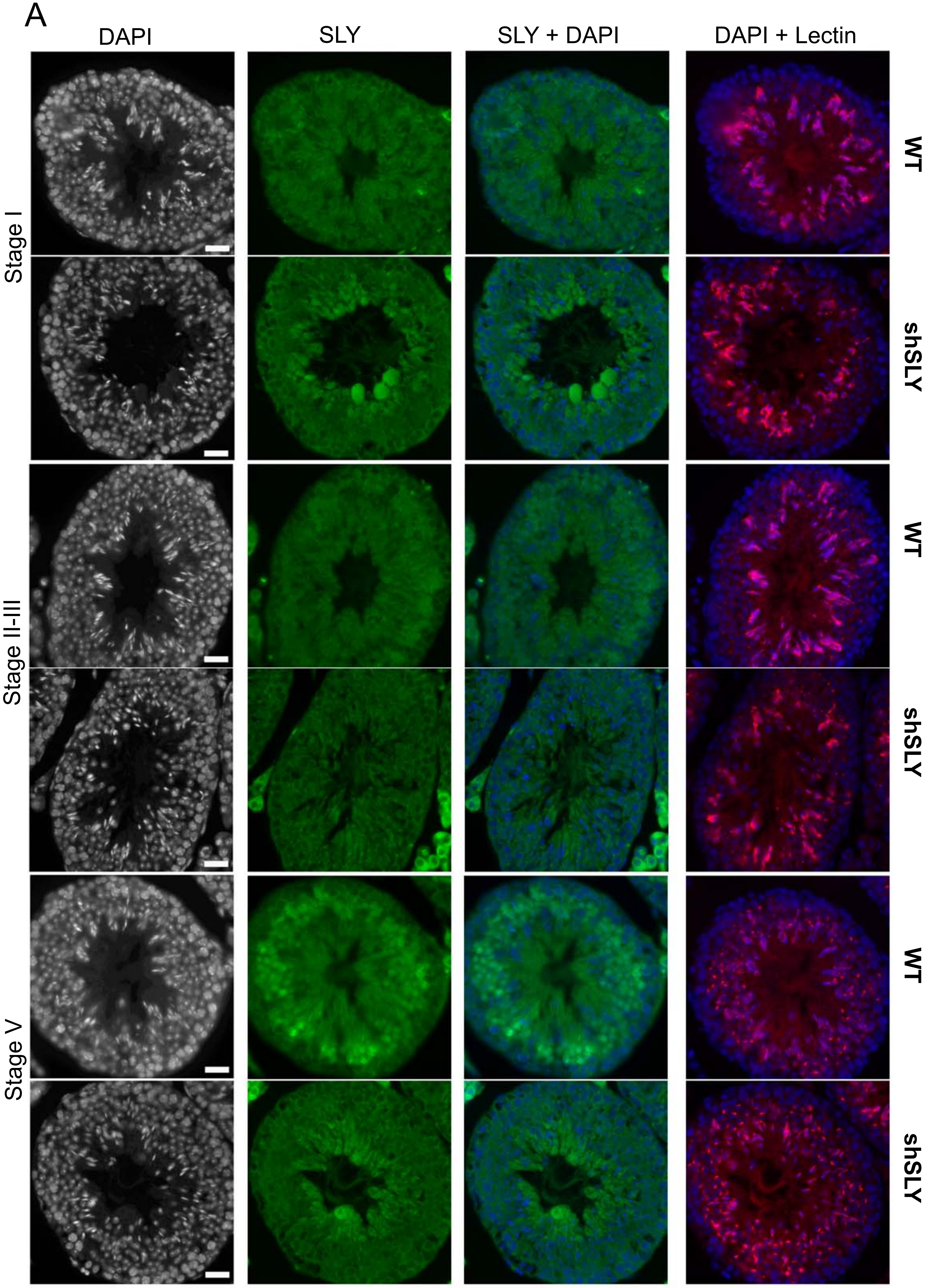
A
DAPI
SLY
SLY + DAPI
DAPI + Lectin
Stage I
Stage II-III
Stage V
WT
shSLY
WT
shSLY
WT
shSLY

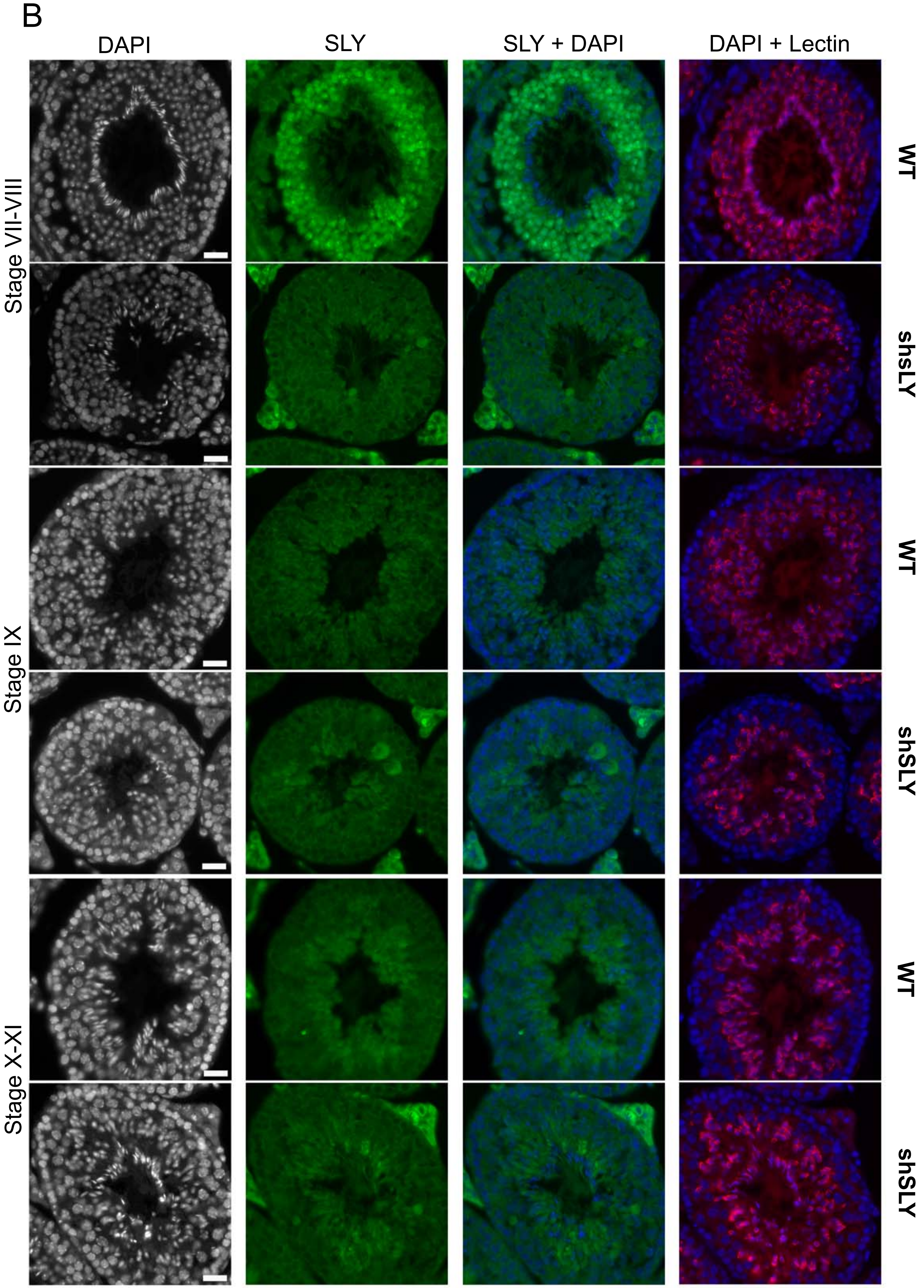
B
DAPI
SLY
SLY + DAPI
DAPI + Lectin
Stage VII-VIII
Stage IX
Stage X-XI
WT
shSLY
WT
shSLY
WT
shSLY

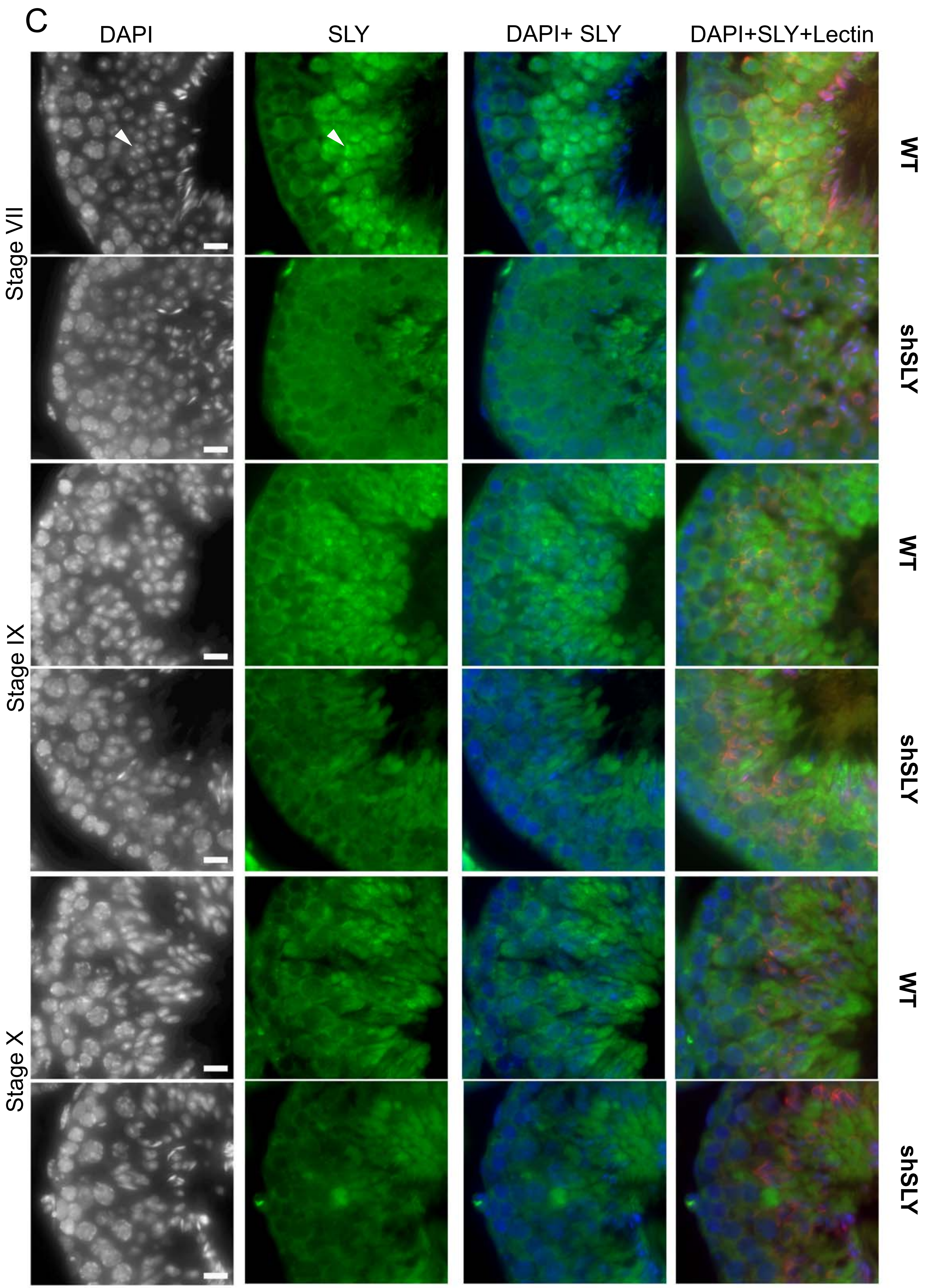

C
DAPI
SLY
DAPI+ SLY
DAPI+SLY+Lectin
Stage VII
Stage IX
Stage X
WT
shSLY
WT
shSLY
WT
shSLY
