# Supplementary material for: The Multicopy Gene Sly Represses the Sex Chromosomes in the Male Mouse Germline after Meiosis
Source: PLoS Biol. 2009 Nov 17;7(11):e1000244. doi: 10.1371/journal.pbio.1000244 (PMC2770110; doi:10.1371/journal.pbio.1000244)
Supplement: Table S1 — Testis weight, sperm number, and sperm motility of sh367 transgenic mice and controls. The mean testis weight value has been calculated from the average testis weight per mouse, for at least seven mice per genotype. Sperm numbers have been scored per cauda epididymis. (0.01 MB PDF) [file pbio.1000244.s012.pdf]

**Category**	**sh367 tsgic**	**controls**
Testis weight (mg)	106.5 ± 5.7	109 ± 5.03
Sperm number (x10^{-6})	4.62 ± 0.95	9.22 ± 2.51
Progressively motile sperm (%)	23.3* ± 5.6	51.9 ± 7.5
Non-progressively motile sperm (%)	21.8* ± 3.1	6.2 ± 3.4
Total motile sperm (%)	45.1 ± 5.4	58.1 ± 5.8

Mean value ± standard errors. * Significantly different from corresponding control ($p < 0.05$; ANOVA).

Table S1. Testis weight, sperm number and sperm motility of sh367 transgenic mice and controls.

The mean testis weight value has been calculated from the average testis weight per mouse, for at least seven mice per genotype. Sperm numbers have been scored per cauda epididymis.
