# Supplementary material for: The Multicopy Gene Sly Represses the Sex Chromosomes in the Male Mouse Germline after Meiosis
Source: PLoS Biol. 2009 Nov 17;7(11):e1000244. doi: 10.1371/journal.pbio.1000244 (PMC2770110; doi:10.1371/journal.pbio.1000244)
Supplement: Table S2 — Breeding data from all sh136 or sh367 transgenic males and negative siblings obtained so far. (0.01 MB PDF) [file pbio.1000244.s013.pdf]

Category	Male identity	Duration of mating (month)	Nb of litters	Total offspring	Average nb of offspring per litter	Average nb of offspring per month
**shSLY tsgic**	sh136 tsgic #1*	4	5	**46**	9.2	11.5
	sh136 tsgic #2	6	0	**0**	N/A	0
	sh136 tsgic #3	3	0	**0**	N/A	0
	sh136 tsgic #4	2	0	**0**	N/A	0
	sh136 tsgic #5	3	0	**0**	N/A	0
	sh136 tsgic #6	3	0	**0**	N/A	0
	sh136 tsgic #7	2	0	**0**	N/A	0
	sh367 tsgic #1*	8	6	**63**	10.5	7.9
	sh367 tsgic #2	12	2	**14**	7.0	1.2
	sh367 tsgic #3	8	1	**9**	9.0	1.1
	sh367 tsgic #4	11	3	**8**	2.7	0.7
	sh367 tsgic #5	2	1	**2**	2.0	1.0
	sh367 tsgic #6	5	0	**0**	N/A	0
**neg sib**	sh136 neg sib#1	6	5	**32**	6.4	5.3
	sh136 neg sib#2	6	5	**48**	9.6	8.0
	sh136 neg sib#3	3	2	**29**	14.5	9.7
	sh136 neg sib#4	3	2	**23**	11.5	7.7
	sh136 neg sib#5	2	1	**12**	12.0	6.0
	sh367 neg sib#1	12	12	**102**	8.5	8.5
	sh367 neg sib#2	11	9	**70**	7.8	6.4
	sh367 neg sib#3	5	5	**39**	7.8	7.8

*Exceptionally fertile shSLY transgenic males.

Table S2. Breeding data from all sh136 or sh367 transgenic males and negative siblings obtained so far.
