# Supplementary material for: The Multicopy Gene Sly Represses the Sex Chromosomes in the Male Mouse Germline after Meiosis
Source: PLoS Biol. 2009 Nov 17;7(11):e1000244. doi: 10.1371/journal.pbio.1000244 (PMC2770110; doi:10.1371/journal.pbio.1000244)
Supplement: Table S3 — List of the primers used in the study. Unless otherwise stated, primers were described previously [6],[16],[58]–[61]. (0.09 MB PDF) [file pbio.1000244.s014.pdf]

Table S3. List of the primers used in the study.

Primers used to produce U6shSLY vectors

Primer name	Sequence
sh136 reverse primer	AAAAAAAGGACGATGACATAAAAGTTTCCCATCTGTGGCTTTACAGAAACTTTTATGTCATCGTCCAAACAAGGCTTTTCTCCAAGGG
sh367 reverse primer	AAAAAAGGATAAATCTGGAGATGACACCCATCTGTGGCTTTACAGTGTCATCTCCAGATTTATCCAAACAAGGCTTTTCTCCAAGGG
shIRR reverse primer	AAAAAACACTACCGTTGTTATAGGTGCCCATCTGTGGCTTTACAGCACCTATAACAACGGTAGTGAAACAAGGCTTTTCTCCAAGGG
U6 Forward primer	ATCCTCTTAAGTCGACGCCGCCATCTCTAG

Real time PCR primers*

Gene name	Primer name	Sequence	Reference
β-actin			[58]
	β-actin-F	GGCACCACACCTTCTACAATG	
	β-actin-R	GTGGTGGTGAAGCTGTAGCC	
Slx (aka *Xmr*)			[6]
	Slx-F	TTCAGATGAAGAAGAAGAGCAGG	
	Slx-R	TCCATATCAAACTTCTGCTCACAC	
Slx-like (aka *AK015913*)			[6]
	Slx-like-F	TTGGAGGACGCTCATTCTG	
	Slx-like-R	ACGACTTGTTGTTGATCATCTCC	
H2AL1 (aka *AK005922*)			[6]
	H2AL1-F	CCAGAGAGGTGAGCTTCCTC	
	H2AL1-R	CAGGCATCATCAGTTGTCATC	
*Actrt1**			
	Actrt1-F	CTCAAAAATGGTCTGCAACAGC	
	Actrt1-R	TCTTGATAGGGGTTCCCTCAAA	
*1700008I05Rik**			
	1700008I05Rik-F	AAAGCCAATTCGTGGAGACAAT	
	1700008I05Rik-R	TGGGAGAGATGCAGAATATCCA	
Ssty1			[6]
	Ssty1-F	AGAAGGATCCAGCTCTCTATGCT	
	Ssty1-R	CCAGTTACCAATCAACACATCAC	
Ssty2			[6]
	Ssty2-F	CAGGTGCCATTCTTACAGGACTAT	
	Ssty2-R	ACCCAGGAACCTATTAAGAAGTCAT	
Asty			[6]
	Asty-F	GRGGAGTAGAACTCATCATC	

	Asty-R	CAGGAGATGACTAACATAGCA	
Orly			[16]
	Orly-S1.f2	TCCCAGTGGTGTATGAAAGG	
	Orly-N1.r	GCCATTGTCTGATGAAAGTACC	
*Zfy2**			
	Zfy2-F	CTTAATTCCAGACATTTTAACTTCCA	
	Zfy2-R	ATCACTTGTTCAAAATGTCCTACATT	
Ubb			[6]
	Ubb-F	GAGGGGTGGCTATTAATTATTCG	
	Ubb-R	CTAAACTTAAATTGGGGCAAGTG	
Acrv1 (aka *SP-10*)			[59]
	Acrv1-F	TGAGTACACCACTTCCAAGCA	
	Acrv1-R	AAGCACATGTGTGGCAATTT	
Protamine 1			[60]
	Protamine1-F	ACAAAATTCCACCTGCTCACA	
	Protamine1-R	GTTTTTCATCGGaCGGTGGC	
Hsf2			[59]
	Hsf2-F	ACCCACACCAACGAGTTCAT	
	Hsf2-R	TGCTCATCCAAGACCAGAAA	
*Oas1b**			
	Oas1b-F	ATCTCATCAGTGGGCGTACC	
	Oas1b-R	CCAGGCATAGACTGTGAGCA	
Sly (global PCR)*			
	Global Sly F	CATTTATAAGACGCTTCACATAAAG	
	Global Sly R1	TCCTCCATGATGGCTCTTTC	
	Global Sly R2	ATTCTCCATGATGGCTCTTTC	

Additional probes for small Northern blot

Probe name	Sequence	
mir-t3 probe	TTACATGAGCTCAAAATCATAC	
mir-t25 probe	AGTAACTGCTACTTCTCTCAGCA	
U6snRNA probe	TTCACGAATTTGCGTGTCATCCTTGCG	[61]

* newly designed primer pairs for Real time PCR were designed to span an intron boundary and the identity of the amplified product was confirmed by sequencing.
